# A synthetic C4 shuttle via the β-hydroxyaspartate cycle in C3 plants

**DOI:** 10.1073/pnas.2022307118

**Published:** 2021-05-17

**Authors:** Marc-Sven Roell, Lennart Schada von Borzyskowski, Philipp Westhoff, Anastasija Plett, Nicole Paczia, Peter Claus, Urte Schlueter, Tobias J. Erb, Andreas P.M. Weber

**Affiliations:** ^a^Institute of Plant Biochemistry, Heinrich Heine University, 40225 Düsseldorf, Germany;; ^b^Department of Biochemistry and Synthetic Metabolism, Max Planck Institute for Terrestrial Microbiology, 35043 Marburg, Germany;; ^c^Cluster of Excellence on Plant Science, Heinrich Heine University, 40225 Düsseldorf, Germany;; ^d^LOEWE Center for Synthetic Microbiology, Philipps-University Marburg, 35043 Marburg, Germany

**Keywords:** photosynthesis, photorespiration, synthetic biology, crop improvement, C4

## Abstract

Plants depend on the enzyme ribulose-1,5-bisphosphate carboxylase/oxygenase (Rubisco) for CO_2_ fixation. However, especially in C3 plants, photosynthetic yield is reduced by formation of 2-phosphoglycolate, a toxic oxygenation product of Rubisco, which needs to be recycled in a high-flux–demanding metabolic process called photorespiration. Canonical photorespiration dissipates energy and causes carbon and nitrogen losses. Reducing photorespiration through carbon-concentrating mechanisms, such as C4 photosynthesis, or bypassing photorespiration through metabolic engineering is expected to improve plant growth and yield. The β-hydroxyaspartate cycle (BHAC) is a recently described microbial pathway that converts glyoxylate, a metabolite of plant photorespiration, into oxaloacetate in a highly efficient carbon-, nitrogen-, and energy-conserving manner. Here, we engineered a functional BHAC in plant peroxisomes to create a photorespiratory bypass that is independent of 3-phosphoglycerate regeneration or decarboxylation of photorespiratory precursors. While efficient oxaloacetate conversion in *Arabidopsis thaliana* still masks the full potential of the BHAC, nitrogen conservation and accumulation of signature C4 metabolites demonstrate the proof of principle, opening the door to engineering a photorespiration-dependent synthetic carbon–concentrating mechanism in C3 plants.

## Significance

Photorespiration is essential for photosynthesis in an oxygen-containing atmosphere. By mass flow, photorespiration is exceeded only by photosynthetic carbon assimilation. Photorespiration, initiated by the oxygenation reaction of Rubisco, is a major constraint on the photosynthetic efficiency of C3 plants and consequently on crop yield. Mitigating the negative effects of photorespiration holds potential for yield increases and contributes to achieving food and energy security for a growing population. This work presents a synthetic bypass to natural photorespiration (i.e., the conversion of photorespiratory glycolate into a C4 compound via a recently discovered microbial glycolate assimilation pathway, the β-hydroxyaspartate cycle [BHAC]). Simultaneous expression of four enzymes of microbial origin in the land plant model *Arabidopsis thaliana* enables efficient glycolate conversion into BHAC products.

Future agriculture must reconcile sustainability with increased productivity to supply global food demands that will have doubled by 2050 (1, 2). To fulfill this goal, agricultural yields will have to increase annually by 2.4%. However, yields currently plateau at a 1% annual increase in major food crops [i.e., maize, rice, and wheat (3, 4)]. In high-yielding crop varieties, both plant architecture and the harvest index—the fraction of total energy in plant biomass contained in the harvestable organs—approach their theoretical limits (5).

Synthetic biology–based approaches are focusing on improving the carbon conversion efficiency of plants that currently only reaches 20% of its theoretical potential (5, 6). Synthetic biology applies engineering principles to biological systems and multiple synthetic biological solutions to improve the carbon conversion efficiency of plants were recently proposed (6, 7). These include pathways for improved CO_2_ fixation (Rubisco based and Rubisco independent), such as the crotonyl-coenzyme A (CoA)/ethylmalonyl-CoA/hydroxybutyryl-CoA cycle (8, 9), photorespiratory bypasses, including the Tartonyl-CoA (TaCo) pathway and a modified 3-hydroxypropionate bicycle (10), as well as synthetic carbon–concentrating mechanisms. Altogether, these proposed solutions showcase the potential of plant synthetic biology to increase productivity and sustainability of future agriculture beyond the realms of natural evolution (7, 11, 12).

Natural carbon-concentrating mechanisms boost carbon fixation by concentrating CO_2_ at the site of Rubisco and have independently evolved in cyanobacteria [carboxysomes (13)], green algae [pyrenoids (14)], and plants [C4 photosynthesis, single-cell C4 photosynthesis, and crassulacean acid metabolism (7)]. In C4 photosynthesis, primary CO_2_ fixation is spatially separated from Rubisco. First, CO_2_ is captured into a C4 acid via phosphoenolpyruvate carboxylase (PEPC) in mesophyll cells, and this C4 acid is then decarboxylated in bundle sheath cells, where Rubisco is located. The increase in the local CO_2_ concentration reduces the oxygenation reaction of Rubisco as well as the subsequent process of photorespiration (15). Consequently, implementation of C4 photosynthesis into C3 plants has received much attention to increase yield in crop plants that experience photorespiration (16, 17).

Another target to improve plant growth is photorespiration itself. During natural photorespiration, the Rubisco oxygenation product 2-phosphoglycolate is recycled back into 3-phosphoglycerate. However, natural photorespiration comes with the loss of up to 30% of previously fixed carbon (18), release of nitrogen, and the dissipation of energy (19), which has led to the engineering of photorespiratory bypasses to mitigate the deleterious effects of photorespiration. In particular, glycolate, formed by dephosphorylation of 2-phosphoglycolate, has been considered an ideal starting metabolite for several photorespiratory bypasses (7, 20). Photorespiratory bypasses that recycle glycolate into 3-phosphoglycerate by the cyanobacterial “glycerate pathway” or oxidize glycolate in the chloroplast have already shown growth benefits in greenhouse-grown *Arabidopsis thaliana* (21, 22) and tobacco and rice in field experiments (23, 24). However, all of these bypasses still release CO_2_, which limits their efficiency compared to natural photorespiration.

**Fig. 1.**
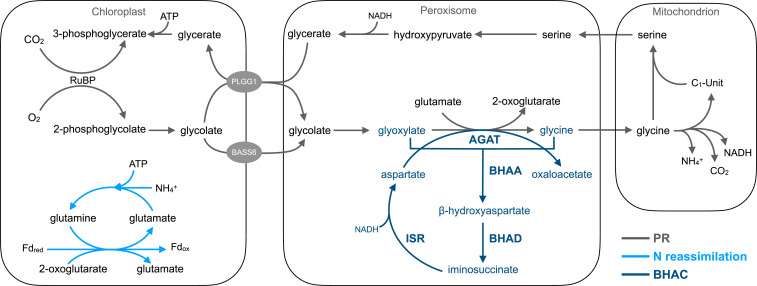
The BHAC as photorespiratory bypass in plant peroxisomes. A schematic representation of plant photorespiration (PR) and photorespiratory nitrogen (N) reassimilation (light blue) and the BHAC (dark blue) AGAT, BHAA, BHAD, ISR, GGT1, ribulose-1,5-bisphosphate (RuBP), plastidial glycolate/glycerate transporter 1 (PLGG1), and bile acid sodium symporter 6 (BASS6).

Recently, the β-hydroxyaspartate cycle (BHAC) was described as primary pathway of glycolate assimilation in marine proteobacteria (25). In this pathway, glycolate is first oxidized into glyoxylate, which is further converted into oxaloacetate (OAA) in four enzymatic steps, the core of the BHAC (Fig. 1). Notably, the BHAC enables the direct formation of a C4 compound from glycolate, without the loss of carbon and nitrogen, which renders the BHAC more efficient than natural photorespiration and all other photorespiration bypasses engineered in vivo so far.

Here, we demonstrate the implementation of the BHAC in *A. thaliana* (*Arabidopsis*) peroxisomes. We validate activity of the BHAC in planta by demonstrating β-hydroxyaspartate (BHA) formation under photorespiratory conditions. Furthermore, we show improved nitrogen conservation through the BHAC, which results in reduced free ammonia levels compared to natural photorespiration. We also determine the metabolic fate of BHAC-derived OAA and outline a strategy to use BHAC-derived OAA to establish a synthetic C4 cycle in C3 plants. Altogether, our proof-of-principle study demonstrates an approach to turn a photorespiratory bypass into a carbon concentrating mechanism by synergistically coupling photorespiration and C4 metabolism. By engineering two of the main targets in primary plant metabolism, this study creates opportunities for improved agricultural productivity in the future.

## Results

### BHAC Implementation in Plant Peroxisomes.

**Fig. 2.**
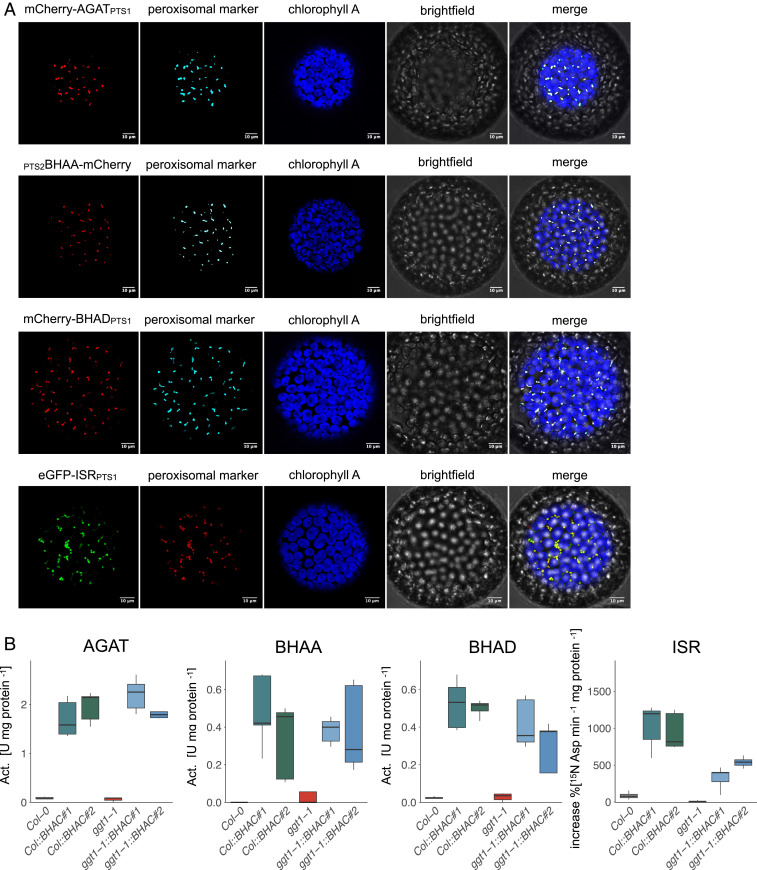
Peroxisomal targeting and enzyme activity of the BHAC. (*A*) Fluorescent fusion constructs for each BHAC enzyme were coinfiltrated with a peroxisomal marker in *N. benthamiana* leaves, and protoplasts were analyzed by confocal microscopy 2 d postinfection. Both the peroxisomal targeting sequence (subscripted) and the fluorescent fusion protein are indicated based on the protein N- or C-terminal position. AGAT, BHAA, BHAD, and ISR. Blue: chlorophyll A autofluorescence, Peroxisomal marker: cyan fluorescent protein or mCherry (only for ISR) with C-terminal PTS1 (Scale bar,10 µm). (*B*) BHAC enzyme activity in *Arabidopsis* mature rosette leave extracts of 4-wk-old air-grown plants. For the ISR assay, the percentual ^15^N label enrichment into aspartate was quantified over time. *n* = 3.

Photorespiratory glycolate is converted to glyoxylate in peroxisomes. Since glyoxylate is the starting substrate of the BHAC, we implemented the BHAC in the peroxisomal matrix. The four BHAC enzymes, aspartate:glyoxylate aminotransferase (AGAT, Enzyme Commission [EC]: 2.6.1.35), β-hydroxyaspartate aldolase (BHAA, EC: 4.1.3.41), β-hydroxyaspartate dehydratase (BHAD, EC: 4.3.1.20), and iminosuccinate reductase (ISR), were targeted to plant peroxisomes by fusion of a peroxisomal target signal (PTS). AGAT, BHAD, and ISR were C terminally fused with PTS1 (26). BHAA was fused N terminally with the PTS2 from *Arabidopsis* citrate synthase 3 [At2g42790 (26)]. Peroxisomal localization of the four BHAC enzymes was confirmed by fluorescence colocalization with a peroxisomal marker in *Nicotiana benthamiana* protoplasts and extrapolated for BHAC implementation in *Arabidopsis* (Fig. 2*A*).

We selected four *Arabidopsis* photosynthetic promoters [Rubisco small subunit 1B, 2B, 3B (27), and chlorophyll A/B–binding protein 1 (28)] to restrict BHAC enzyme expression to photosynthetic tissue (*SI Appendix*, Fig. S1). Furthermore, we hypothesized that reduced conversion of glyoxylate to glycine would enhance metabolic flux through the BHAC. Besides *Arabidopsis* wild-type *Col-0* (WT), we therefore selected the photorespiratory *ggt1-1* mutant as background for BHAC implementation. The *ggt1-1* mutant is deficient in the peroxisomal glutamate glyoxylate aminotransferase 1 (29) and shows a strong photorespiratory phenotype, which allowed us to screen for the function of the BHAC via a convenient visual readout.

In total, 14 and 11 primary transformants that harbor the complete transfer DNA insertion were identified in the WT and *ggt1-1* background, respectively. Out of these two independent lines, each were established in the WT (*Col::BHAC #1* and *#2*) and *ggt1-1* background, respectively (*ggt1-1::BHAC #1* and *#2*, *SI Appendix*, Fig. S1), based on immunoblot analysis to verify expression of all four BHAC enzymes in the transgenic lines (*SI Appendix*, Fig. S1). We quantified activity of each BHAC enzyme in mature rosette leaf extracts of 4-wk-old air-grown plants by enzyme activity assays (Fig. 2*B*). AGAT was highest in both WT and *ggt1-1* backgrounds compared to BHAA and BHAD activity (Fig. 2*B*). Iminosuccinate is a labile product formed by BHAD (25). To demonstrate functional expression of ISR, we therefore quantified the rate of ^15^N incorporation into L-aspartate, which confirmed ISR activity in BHAC plants (Fig. 2*B*). In summary, these experiments confirmed the successful expression of all enzymes in planta.

### Peroxisomal BHAC Functions as Photorespiratory Bypass.

**Fig. 3.**
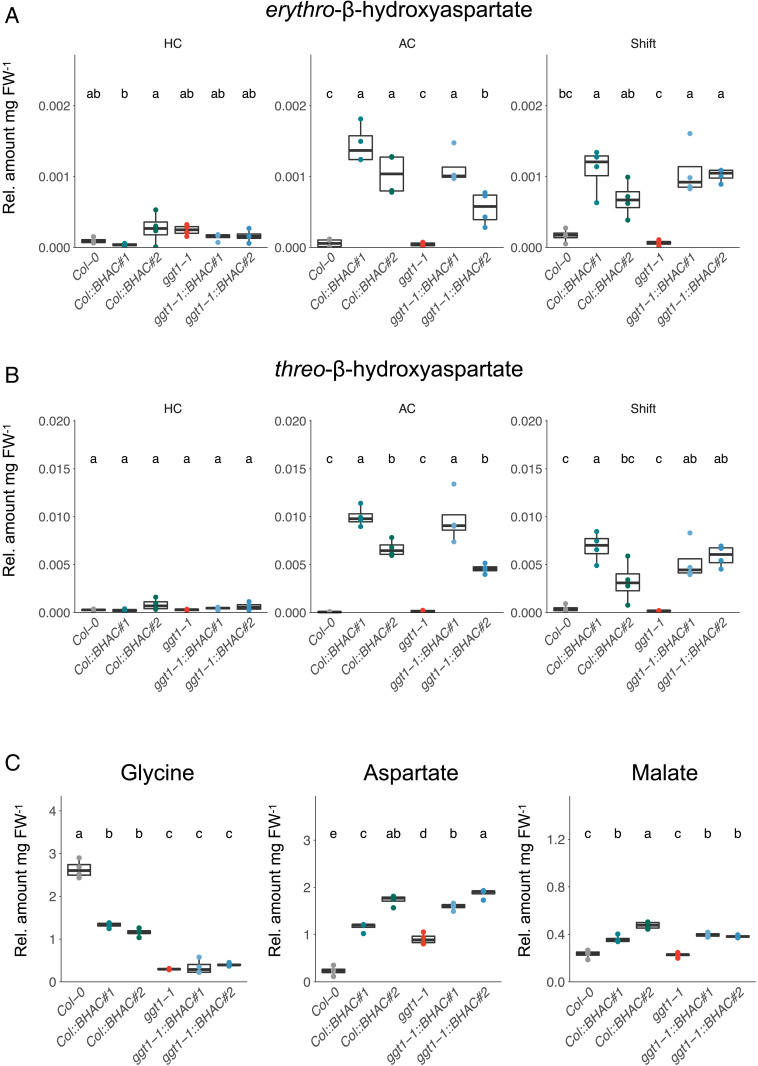
The BHAC functions as photorespiratory bypass. (*A* and *B*) Relative metabolite levels per mg fresh weight (FW) of in vivo *erythro*-β-hydroxyaspartate (*A*) and *threo*-β-hydroxyaspartate (*B*) formation in BHAC plants. Green tissue of 14-d-old seedlings was harvested in the middle of the light phase. The plants were grown either in CO_2_-enriched air (3,000 ppm CO_2_, HC), ambient air (400 ppm CO_2_, AC), or shifted from HC to AC 3 d prior to harvest (Shift). (*C*) Relative metabolite levels per mg FW of glycine, aspartate, and malate in BHAC plants grown in ambient air. Each box–whisker plot represents the 25th and 75th percentiles and whiskers the 10th and 90th percentile. The median is indicated as a crossbar. One-way ANOVA with a post hoc Tukey honest significant difference test was used for statistical analysis. The different letters indicate significant differences between genotypes at *P* < 0.05. *n* = 4.

Next, we verified that the peroxisomal BHAC functions as photorespiratory bypass by steady-state metabolomics on green tissue of 14-d-old seedlings either grown in CO_2_-enriched air (3,000 ppm CO_2_, high carbon, HC), ambient air (400 ppm CO_2_, ambient carbon, AC), or shifted from CO_2_-enriched to ambient air 3 d before sampling (Shift, Fig. 3). Our metabolomics analysis included the BHAC intermediates BHA and glycine; malate, produced by reduction of BHAC-derived OAA by peroxisomal NAD–dependent MDH (30), as well as aspartate, which can be regarded both as BHAC intermediate and product of OAA transamination (Fig. 3).

BHA is a unique metabolite of the BHAC and not naturally present in *Arabidopsis* (Fig. 3 *A* and *B*). We analyzed analytical standards of BHA diastereomers via gas chromatography–time-of-flight mass spectrometry (GC/MS Q-TOF) to annotate BHA according to the electron impact mass spectral fragmentation pattern (*SI Appendix*, Fig. S2). As expected, BHA-specific fragments were neither detected in WT nor in *ggt1-1* controls under all conditions tested. In contrast, BHA could be detected in plants carrying the BHAC (*SI Appendix*, Fig. S3). However, relative quantification revealed that BHA was only detectable when plants were grown in ambient air or shifted from CO_2_-enriched to ambient air but not in CO_2_-enriched air (Fig. 3 *A* and *B*). This confirmed function of the BHAC in planta and suggested that BHA formation is exclusively linked to photorespiratory conditions.

Glycine levels decreased twofold in both *Col::BHAC* lines, which is consistent with glycine conversion into BHA by BHAA under photorespiratory conditions in ambient air (Fig. 3*C*). In the *ggt1-1* mutant total glycine levels were 10-fold lower compared to WT (29) and remained unaltered in *ggt1-1::BHAC* plants (Fig. 3*C*) despite partial restoration of peroxisomal glyoxylate to glycine conversion through a promiscuous aminotransferase activity of AGAT in the *ggt1-1* mutant (Fig. 3*C*). In line with BHAC activity, aspartate and malate levels were elevated sixfold and twofold, respectively, in ambient air–grown BHAC plants of both background genotypes compared to WT (Fig. 3*C*). Together, the formation and accumulation of BHAC-specific metabolites exclusively in photorespiratory conditions demonstrated that the peroxisomal BHAC indeed functions as photorespiratory bypass.

### The BHAC Reshapes Carbon and Nitrogen Metabolism.

**Fig. 4.**
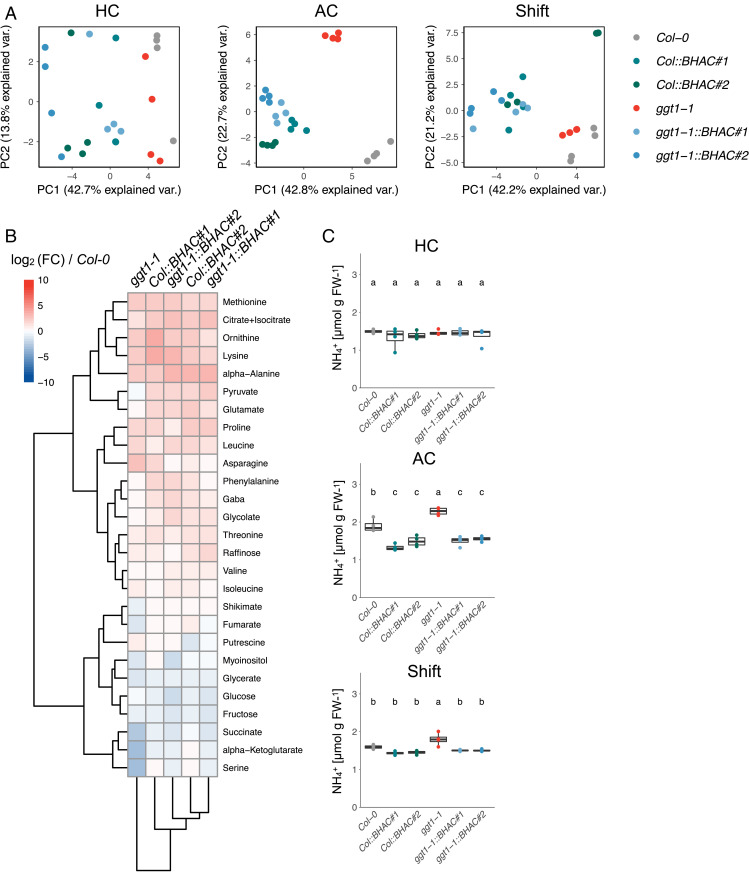
The BHAC reshapes the plant metabolome. Metabolite profiles were generated using green tissue of 14-d-old seedlings grown either at 3,000 (HC), 400 (AC), or shifted from 3,000 to 400 ppm CO_2_ 3 d (Shift) prior to harvest at the middle of the light phase. (*A*) Principal component analysis of the metabolome profiles. (*B*) Metabolome profiles of AC grown plants. The log2 fold change (FC) was calculated compared to wild-type *Col-0* and clustered based on Pearson correlation. (*C*) Quantification of free ammonium in BHAC plants. Each box–whisker plot represents the 25th and 75th percentiles and whiskers the 10th and 90th percentile. The median is indicated as a crossbar. One-way ANOVA with a post hoc Tukey honest significant difference test was used for statistical analysis. The different letters indicate significant differences between genotypes at *P* < 0.05. *n* = 4 biological replicates measured in technical triplicates.

To better understand the metabolic implications of the BHAC, we generated metabolite profiles for all four BHAC lines (BHAC plants) and their background genotypes at different CO_2_ concentrations (HC, AC, and Shift; *SI Appendix*, Fig. S4). Growth condition–dependent principal component analysis revealed that the metabolic profiles of BHAC plants are clearly distinct from their background genotypes (Fig. 4*A*) and that all BHAC plants cluster together, independent of their genetic background under photorespiratory conditions (AC or Shift; Fig. 4*A*). Notably, we did not observe such clustering of genotypes under HC (Fig. 4*A*), which is consistent with the observation that the BHAC is only active under photorespiration.

We further focused on the metabolite profile of BHAC plants in comparison to the WT and *ggt1-1* mutant backgrounds grown under photorespiratory conditions in ambient air (Fig. 4*B*). In plant photorespiration mitochondrial glycine decarboxylase is the major hub of carbon and nitrogen losses (19, 31). Nitrogen conservation by the BHAC is assumed to prevent mitochondrial ammonia release and avoid chloroplastic nitrogen reassimilation by glutamine synthase. Consequently, cellular free ammonia levels were reduced on average by 20% in ambient air–grown BHAC plants compared to WT under photorespiratory conditions (Fig. 4*C*). Furthermore, ambient air–grown BHAC plants accumulated soluble amino acids that are either involved in the urea cycle (glutamate and ornithine) or depend on OAA-derived carbon skeletons (lysine and methionine; Fig. 4*B*).

Besides peroxisomal reduction to malate, three further routes of OAA assimilation are theoretically possible that are all coupled to the direct export of OAA from the peroxisome (32). Cytosolic phosphoenolpyruvate carboxykinase 1 (PCK1) could decarboxylate OAA to phosphoenolpyruvate [PEP (33)]. PEP is then used either for gluconeogenesis or converted into pyruvate by pyruvate kinase [PK (34)]. The accumulation of pyruvate strongly indicated that the cytosolic PK route is active in BHAC plants and that PEP is not channeled into gluconeogenesis, which was supported by reduced glucose and fructose levels in the same plants (Fig. 4*B*). In addition to cytosolic decarboxylation, OAA could also be transported into mitochondria, where it could fuel the mitochondrial tricarboxylic acid (TCA) cycle. Accumulation of citrate in ambient air–grown BHAC plants suggested that this route was also active, eventually in combination with an increased flux of pyruvate into the TCA cycle (Fig. 4*B*).

To further validate that reshaping of the metabolome in BHAC plants is caused by an active BHAC and not AGAT alone, we complemented the *ggt1-1* mutant with AGAT under control of the chlorophyll A/B–binding protein 1 promoter, which was also used for AGAT expression in *ggt1-1:BHAC* plants (*SI Appendix*, Fig. S5). Steady-state metabolomics on plants grown under photorespiratory conditions in ambient air revealed that AGAT expression was not sufficient to cause the distinct metabolome signature of BHAC plants. Instead, AGAT expression only restored canonical photorespiration, probably by utilizing aspartate as amino donor for the peroxisomal transamination reaction (*SI Appendix*, Fig. S5).

In summary, these experiments showed that the BHAC is active under photorespiratory conditions and reshapes the metabolome in plants by altering nitrogen metabolism (amino acid accumulation and free ammonia reduction) and OAA utilization in the cytosol and/or mitochondrial TCA cycle.

### The BHAC Reduces Plant Growth by Impaired Photosynthesis in the WT Background.

**Fig. 5.**
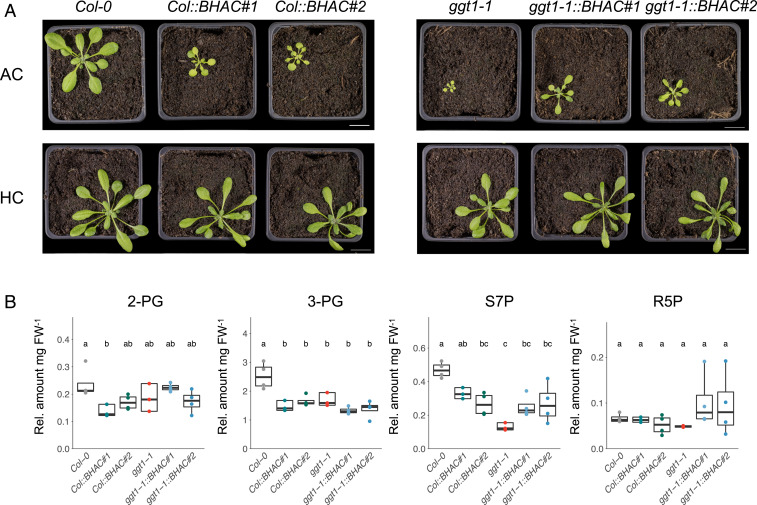
The BHAC reduces plant growth in air by impaired photosynthesis. (*A*) Representative images of BHAC plants in ambient air (400 ppm CO_2_, AC) or CO_2_-enriched air (3,000 ppm CO_2_, HC). The images were taken 28 d after transfer to light (Scale bar, 2 cm). (*B*) Relative levels of phosphorylated metabolites per mg fresh weight (FW) of 2-phosphoglycolate (2-PG), 3-phosphoglycerate (3-PG), seduheptulose-7-phosphate (S7P), and ribulose/ribose-5-phosphate (R5P) in BHAC plants grown in ambient air. Each box–whisker plot represents the 25th and 75th percentiles and whiskers the 10th and 90th percentile. The median is indicated as a crossbar. One-way ANOVA with a post hoc Tukey honest significant difference test was used for statistical analysis. The different letters indicate significant differences between genotypes at *P* < 0.05. *n* ≥ 3.

Despite carbon and nitrogen conservation by the BHAC, *Col::BHAC* plants are reduced in growth compared to WT controls in ambient air (Fig. 5*A* and *SI Appendix*, Fig. S6). Rosettes of 4-wk-old air-grown *Col::BHAC* plants are decreased by 70% in area and 50% in diameter (*SI Appendix*, Fig. S7). However, BHAC implementation in the *ggt1-1* mutant partially suppressed the photorespiratory phenotype of the mutant (Fig. 5*A*) and growth was comparable to *Col::BHAC* plants (*SI Appendix*, Fig. S7). In CO_2_-enriched air, growth of BHAC plants was not altered compared to the background genotype (Fig. 5*A* and *SI Appendix*, Fig. S7).

In order to test the hypothesis that reduced plant growth is caused by suppressed photorespiratory 3-phosphoglycerate regeneration in BHAC plants, we analyzed steady-state levels of phosphorylated intermediates in green tissue of 14-d-old air-grown plants (Fig. 5*B* and *SI Appendix*, Fig. S8). Whereas 2-phosphoglycolate levels were not significantly altered between all genotypes, the 3-phosphoglycerate levels were reduced in BHAC plants compared to WT and notably very similar to the *ggt1-1* mutant, impaired in photorespiratory 3-phosphoglycerate regeneration (Fig. 5*B*). In addition, in BHAC plants, 3-phosphoglycerate levels were similar to the *ggt1-1* mutant, impaired in photorespiratory 3-phosphoglycerate regeneration (Fig. 5*B*). Furthermore, sedoheptulose-7-phosphate, an intermediate of the Calvin-Benson-Bassham cycle (CBBC), showed reduced levels in BHAC plants, whereas ribose/ribulose-5-phosphate levels were not altered (Fig. 5*B*). We directly assessed the photosynthetic capacity of BHAC plants and generated A/C_i_ curves, the rate of CO_2_ assimilation (A) in relation to intercellular CO_2_ concentration (C_i_) under saturating light (1,000 µmol ⋅ s^−1^ ⋅ m_2_^−2^), by leaf gas exchange measurements of 6-wk-old ambient air–grown plants (*SI Appendix*, Fig. S9). Based on the A/C_i_ curve, we determined the CO_2_ compensation point (CCP), a net quotient of zero for photosynthetic CO_2_ assimilation and respiratory CO_2_ release (35). *Col::BHAC* plants displayed an increased CCP compared to WT (Table 1). The *ggt1-1* mutant itself has a higher CCP, and the BHAC did not significantly alter the CCP in this photorespiratory mutant (Table 1). In addition, the maximal assimilation rate at saturated CO_2_ (A_max_) and the initial slope as in vivo measure of the carboxylation efficiency were inferred from the A/C_i_ curves (36). Compared to WT, both A_max_ and initial slope were reduced by 25% in *Col::BHAC* plants, while they behaved comparably in *ggt1-1::BHAC* plants (Table 1). Remarkably, neither dark respiration, assessed by light response curve measurements at constant CO_2_ (400 µbar) nor the CCP under low-oxygen conditions (4% O_2_) were altered across all genotypes, except a slightly elevated CCP in *ggt1-1::BHAC#2* plants (Table 1 and *SI Appendix*, Fig. S9). Western blot analysis of the Rubisco large subunit and the Rieske–Fe protein demonstrated that the reduced initial slope and A_max_ in BHAC plants was not caused by altered protein content of these enzymes (*SI Appendix*, Fig. S10).

**Table 1.** Physiological parameters of BHAC plants

	CCP (µmol ⋅ mol^−1^)	A_max_ (µmol CO_2_ m^−2^ ⋅ s^−1^)	A/C_i_ slope (mol ⋅ m^−2^ ⋅ s^−1^)	R_dark_ (µmol CO_2_ m^−2^ ⋅ s^−1^)	CCP ^4%^ ^O^_2_ (µmol ⋅ mol^−1^)
*Col-0*	61.17 ± 2.5^b^	16.52 ± 2.2^a^	0.045 ± 0.005^a^	−1.07 ± 0.2^a^	15.8 ± 3.3^b^
*Col::BHAC#1*	71.20 ± 3.3^ab^	12.36 ± 1.5^b^	0.035 ± 0.003^ab^	−0.82 ± 0.1^a^	17.3 ± 0.7^ab^
*Col::BHAC#2*	73.53 ± 3.7^a^	10.82 ± 2.2^b^	0.031 ± 0.007^bc^	−0.81 ± 0.1^a^	19.7 ± 1.7^ab^
*ggt1-1*	80.20 ± 4.2^a^	13.52 ± 1.0^ab^	0.021 ± 0.004^c^	−0.94 ± 0.2^a^	19.7 ± 0.6^ab^
*ggt1-1::BHAC#1*	77.98 ± 3.6^a^	11.8 ± 1.1^b^	0.030 ± 0.002^bc^	−0.78 ± 0.2^a^	19.8 ± 2.2^ab^
*ggt1-1::BHAC#2*	77.58 ± 9.8^a^	10.35 ± 1.4^b^	0.026 ± 0.005^bc^	−1.02 ± 0.3^a^	22.8 ± 2.4^a^

The CCP, A_max_, and the slope were determined from the A/C_i_ curves (*SI Appendix*, Fig. S8). Dark respiration (R_dark_) was determined from the light response curves, and the O_2_ dependency of the CCP was determined at low oxygen concentrations (4%). One-way ANOVA with a post hoc Tukey honest significant difference test was used for statistical analysis. The different superscript letters indicate significant differences between genotypes at *P* < 0.05. *n* ≥ 3. Shown are mean ± SD.

## Discussion

An estimated loss of 30% photosynthetically fixed carbon define photorespiration as a limiting factor of plant growth. However, photorespiratory bypasses can address this issue and improve plant yield (6, 12). The recently described BHAC, naturally found in marine proteobacteria, allows the direct conversion of glyoxylate into OAA, providing options to assimilate photorespiratory glyoxylate without the loss of carbon and nitrogen (25). Here, we report on engineering a functional BHAC in *Arabidopsis* peroxisomes to demonstrate a photorespiratory bypass independent of 3-phosphoglycerate regeneration or decarboxylation of a photorespiratory precursor.

Redirecting the metabolic flux toward a synthetic pathway was demonstrated by combining transcriptional suppression of the plastidial glycolate/glycerate transporter 1 with chloroplastic glycolate decarboxylation in field-grown tobacco plants (23). Similarly, implementing the BHAC in the *ggt1-1* mutant to push pathway flux improved plant growth compared to the mutant background. In plant peroxisomes, the BHAC bypasses the mitochondrial glycine decarboxylase complex that would otherwise release ammonia during photorespiration (Fig. 1). Ammonia reassimilation by passive transport to the chloroplast and refixation by the glutamine synthetase 2/ferredoxin–dependent glutamine:oxoglutarate aminotransferase complex (GS2/Fd-GOGAT) is an integral part of photorespiration (19, 37). Based on the metabolite profiles, we hypothesize three metabolic adaptations that compensate the impaired nitrogen shuttle in BHAC plants. A general response upon impaired GS2/Fd-GOGAT–dependent nitrogen assimilation is the use of cytosolic glutamine synthetases and glutamate dehydrogenases (38). Furthermore, excess nitrogen is stored in the urea cycle (39) and the ornithine–citrulline shuttle that might underpin mitochondrial chloroplastic nitrogen exchange (40). Finally, BHAC-derived OAA can be directly converted into aspartate by aspartate aminotransferase (41). Produced aspartate is used for chloroplastic de-novo biosynthesis of amino acids dependent on C4-carbon skeletons, in particular lysine, threonine, and methionine, that accumulate in BHAC plants [Fig. 4 (42, 43)]. This implies that the BHAC functions as a nitrogen-conserving pathway and allows rerouting of photorespiratory glycolate into amino acids.

In contrast to previous photorespiratory bypasses (21–24), the BHAC also alters the carbon stoichiometry of photorespiration. C3 plants depend on the regeneration of 3-PGA by photorespiration, which is exemplified by the strong phenotype of several photorespiratory mutants (29, 44–46). In BHAC plants, glycolate is directly converted into OAA, and the reduced carboxylation efficiency (initial slope) and A_max_ are caused by lowered metabolic flux in the 3-phosphoglycerate–regenerating branch of photorespiration in the WT background and comparable to the photorespiratory *ggt1-1* mutant (Fig. 5*B* and Table 1).

Whereas previously described photorespiratory bypasses release four CO_2_ molecules per two molecules of glycolate (22, 23), the BHAC is carbon neutral and maximally releases one CO_2_ molecule in case OAA is decarboxylated into PEP (25). We note that the introduction of the BHAC into plant peroxisomes did not improve the CCP compared to WT (Table 1). This suggests that either streamlining OAA assimilation or reintegration of the produced C3-intermediate PEP and/or CO_2_ into the CBBC will be the key to achieve the full potential of the BHAC. At current stage, however, pleiotropic effects of diffuse OAA metabolism by several routes (amino acid biosynthesis, TCA cycle, and PEP/pyruvate metabolism) and reduced 3-phosphoglycerate regeneration that lowers the photosynthetic efficiency likely mask the full potential of the BHAC (Fig. 4). Integrating the BHAC into kinetic- and genome-scale metabolic models will help to identify further engineering targets (20, 47–49). Finally, the construction of a synthetic C4 cycle based on BHAC-derived OAA, either in a single cell or spatially separated cycle between mesophyll- and bundle-sheath cells would allow to enhance carbon assimilation in plants (17, 50) (*SI Appendix*, Fig. S11). We note that using photorespiration as source of the synthetic C4 cycle would circumvent the need to establish PEPC-dependent CO_2_ fixation in C3 plants, make an ATP-dependent regeneration of PEP dispensable, and ultimately conserve energy.

In summary, this work on engineering a functional BHAC into *Arabidopsis* is a starting point to turn a photorespiratory bypass into a synthetic C4 cycle, constituting a promising approach toward creating higher crop yields in the future.

## Materials and Methods

### Chemicals.

D-*erythro*-BHA ([2R,3S]-β-hydroxyaspartate) was custom synthesized and determined to be >95% pure by NMR analysis (NewChem). DL-*threo*-BHA was purchased as racemic mixture (Sigma Aldrich).

### Plasmid Construction.

BHAC genes were codon optimized for expression in *A. thaliana* by gene synthesis (ThermoFisher Scientific) and matured for golden-gate cloning. All plasmids were generated with the MoClo tool kit, including vector backbones and genetic parts (51). Plasmids were sequenced by Sanger sequencing (Microsynth). Plasmids and primers used in this study are listed in *SI Appendix*, Tables S1 and S2, respectively.

### BHAC Enzyme Activity Assays.

BHAC enzyme activity was measured in total leaf protein extracts from 4-wk-old air-grown *Arabidopsis* plants. Purified recombinant BHAC enzymes were produced as described in ref. 25. The reaction mixture to assay AGAT activity contained 100 mM potassium phosphate buffer (pH = 7.5), 0.1 mM PLP, 0.2 mM NADH, 5 mM glyoxylate, 20 mM aspartate, 25 µL of leaf extract, and 8.75 µg NAD-dependent malate dehydrogenase (Sigma Aldrich). The reaction mixture to assay BHAA activity contained 100 mM potassium phosphate buffer (pH = 7.5), 0.1 mM PLP, 0.2 mM NADH, 0.5 mM MgCl_2_, 5 mM glyoxylate, 10 mM glycine, 25 µL of leaf extract, and 7 µg purified BHAD and 7 µg purified ISR enzyme. The reaction mixture to assay BHAD activity contained 100 mM potassium phosphate buffer (pH = 7.5), 0.1 mM PLP, 0.2 mM NADH, 2 mM *erythro*-BHA, 25 µL of leaf extract, and 7 µg purified ISR enzyme. The reaction mixture to assay ISR activity contained 100 mM potassium phosphate buffer (pH = 7.5), 0.1 mM PLP, 0.2 mM NADH, 5 mM glyoxylate, and 10 mM ^15^N-glycine, 50 µL of leaf extract, and 7 µg purified BHAA and BHAD enzyme. The formation of ^15^N-aspartate by ISR activity was confirmed by liquid chromatography with tandem mass spectrometry (LC-MS/MS). A detailed description of the LC-MS/MS method is provided in the *SI Appendix*, Text.

### Plant Material and Cultivation Conditions.

The *A. thaliana* ecotype *Col-0* and the *ggt1-1* mutant (29), deficient in the peroxisomal glutamate:glyoxylate aminotransferase 1 (GGT1) were used as reference backgrounds. Seeds were surface sterilized using the vapor-phase sterilization method (52). Seeds were grown on half-strength Murashige and Skoog medium (pH = 5.7) supplemented with 0.8% (wt/vol) agar. Seeds were cold stratified for 2 d at 4 °C. After germination, seedlings were grown for 14 d at 100 µmol m^−2^ ⋅ s^−1^ light intensity at atmospheric CO_2_ concentration (400 ppm) or in CO_2_-enriched air (3,000 ppm) in 12-h light/12-h dark photoperiod prior transfer to soil.

### Metabolite Profiling.

For metabolite profiling, green tissue of 14-d-old seedlings was harvested by immediate quenching with liquid nitrogen at the middle of the light phase. A total of 50 mg leaf material was extracted as previously described (53) and analyzed by GC/MS Q-TOF (Agilent). For ion chromatography–mass spectrometry (IC/MS, Thermo Fisher Scientific), tissue was extracted as previously described (54). For relative quantification, metabolite peak areas were normalized to the internal extraction standard and the material fresh weight. A detailed description of the GC/MS Q-TOF and IC/MS methods and the respective data analysis is provided in the *SI Appendix*, Text.

### Gas Exchange Measurements.

Mature rosette leaves of 6-wk-old air-grown plants were used for gas exchange measurements. Measurements were performed using a LICOR6800 (Licor Bioscience) with a flow set to 300 µmol s^−1^, saturating light intensity of 1,000 µmol m^−2^ ⋅ s^−1^, leaf temperature of 25 °C, and a vapor pressure deficit below 1.5 kPa. A/C_i_ curves were measured via stepwise changes in external CO_2_ supply ranging from 0 to 1,600 µbar. From the A/C_i_ curves the CO_2_ compensation point was determined as x-intercept (36). The initial slope of the A/C_i_ curve was calculated in the linear range between 0 and 200 µbar external CO_2_, and the maximal assimilation rate (A_max_) above 1,000 µbar CO_2_ was determined. Dark respiration was determined by light response measurements at constant external CO_2_ of 400 µbar, and light intensities were stepwise reduced from 1,600 to 0 µmol m^−2^ ⋅ s^−1^. The O_2_ dependency of the CO_2_ compensation point was determined by stepwise reducing the external CO_2_ from 400 to 10 µbar under an applied N_2_-pressured air mixture at a ratio of 4:1, providing an estimated O_2_ concentrations of 4%. The gas mixture was realized with the help two variable air flowmeters. The leaves were always allowed to adjust to the O_2_ conditions for ∼15 min.

## Supplementary Material

Supplementary File

Supplementary File

Supplementary File

Supplementary File

Supplementary File

Supplementary File

Supplementary File

Supplementary File

Supplementary File

Supplementary File

Supplementary File

## Data Availability

Data analysis was performed in R. For analysis of gas exchange measurements, the “plantecophys” package was used (55). The data are summarized in Datasets S1–S10. All other study data are included in the article and/or supporting information.

## Change History

September 27, 2021: Table 1 and the author contributions have been updated; please see accompanying Correction for details.
