# Supplementary material for: A synthetic C4 shuttle via the β-hydroxyaspartate cycle in C3 plants
Source: Proc Natl Acad Sci U S A. 2021 May 17;118(21):e2022307118. doi: 10.1073/pnas.2022307118 (PMC8166194; doi:10.1073/pnas.2022307118)
Supplement: Supplementary File [file pnas.2022307118.sapp.pdf]

PNAS
www.pnas.org

Supplementary Information for

A synthetic C4 shuttle via the β-hydroxyaspartate cycle in C3 plants

Marc-Sven Roell,[a] Lennart Schada von Borzykowski,[c] Philipp Westhoff,[a,b], Anastasija Plett, [a,b] Nicole Paczia,[c] Peter Claus,[c] Urte Schlueter,[a] Tobias J. Erb,[c,d] Andreas P.M. Weber [a,b,1]

Andreas P.M. Weber
Email: andreas.weber@hhu.de

This PDF file includes:

Supplementary text
Figures S1 to S11
Tables S1 to S2
SI References

Supplementary Information Text

Genetic transformation of *Arabidopsis thaliana*

T-DNA constructs were introduced into *Agrobacterium tumefaciens* strain GV3101::pMP90 (1) and transgenic *Arabidopsis thaliana* plants were generated via *Agrobacterium*-mediated transformation and floral dipping (2). Homozygous T3 plants were used for further analysis. Transgenic plants were selected on half-strength Murashige and Skoog agar plates containing kanamycin (50 µg ml^{-1}) as selection marker. Homozygous T3 plants were used for physiological analysis.

Transient expression in *Nicotiana benthamiana* and protoplast isolation

Overnight grown *Agrobacterium tumefaciens* GV3101::pMP90 cells, carrying the T-DNA construct were diluted in infiltration medium (10 mM $MgCl_2$, 10 mM MES [pH 5.7], 100 µM acetosyringone) to an OD_{600} of 0.4. Leaves of four-weeks-old greenhouse-grown *Nicotiana benthamiana* plants were infiltrated using a syringe without a needle. For fluorescence co-localization analysis peroxisomal marker T-DNA constructs were co-infiltrated, expressing either cyan fluorescent protein or mCherry, C-terminally fused with peroxisomal target signal 1 (PTS1). Two days post infection, leaves were sliced into small pieces, vacuum-infiltrated with protoplast digestion solution (1.5% [w/v] cellulase R-10, 0.4% [w/v] macerozyme R-10, 0.4 M mannitol, 20 mM KCl, 20 mM MES [pH 5.7], 10 mM $CaCl_2$, 0.1% [w/v] bovine serum albumin) and incubated for 2 hours at 28°C. Sedimented protoplasts were resuspended in W5 solution (154 mM NaCl, 125 mM $CaCl_2$, 5 mM KCl, 2 mM MES [pH5.7]) and analyzed by confocal laser scanning microscopy.

Confocal laser scanning microscopy

Zeiss LSM780 confocal microscope and Zeiss ZEN software (Zeiss) was used for confocal laser scanning microscopy. Excitation/emission wavelengths were as followed: mCherry (561 nm/580 to 625), cyan fluorescent protein (450 nm/510 to 550) nm), green fluorescent protein (488 nm/490 to 550 nm), chlorophyll A (488 nm/640 to 710 nm). Images were processed with Fiji (3).

Enzyme activity assays

Total leaf protein was isolated from four-weeks-old air-grown *Arabidopsis* plants. Leaf material was frozen in liquid nitrogen and ground, using glass beads and mixermill and resuspended in 700 µl extraction buffer (50 mM potassium phosphate [pH 7.5], 5 mM $MgCl_2$, 1 mM EDTA, 0.1% [v/v] Triton-X 100). After centrifugation for 10 min at 12,000 rpm at 4°C, 25 µl of the supernatant was used for enzyme assays. All assays were carried out at 30°C in a total volume of 300 µl. The oxidation of NADH was followed at 340 nm on a Cary 60 UV-Vis photospectrometer (Agilent) in quartz cuvettes with a path length of 10 mm (Hellma Analytics).

Glutamate:glyoxylate activity measurements were performed based on AGAT assays as described. Aspartate was replaced by 50 mM glutamate.

To take samples for LC-MS/MS analysis, the reaction volume of the assay was increased to 600 µl. 90 µL aliquots were taken after 0, 1, 2 and 3 minutes and the reaction was immediately stopped by addition of formic acid (4% final concentration). The samples were centrifuged at 17,000 x g and

4°C for 15 min and the supernatant was subsequently used for LC-MS analysis. Quantitative determination of ^{15}N-aspartate (Asp-^{15}N) and Aspartate (Asp) was performed using a LC-MS/MS. The chromatographic separation was performed on an Agilent Infinity II 1290 HPLC system using a ZicHILIC SeQuant column (150 × 2.1 mm, 3.5 µm particle size, 100 Å pore size) connected to a ZicHILIC guard column (20 × 2.1 mm, 5 µm particle size, Merck KgAA) at a constant flow rate of 0.3 ml/min with mobile phase A being 0.1% formic acid in 99:1 water:acetonitrile (Honeywell, Morristown, New Jersey, USA) and phase B being 0.1% formic acid in 99:1 acetonitrile:water (Honeywell, Morristown, New Jersey, USA) at 25°C. The injection volume was 5 µl. The mobile phase profile consisted of the following steps and linear gradients: 0 – 5 min from 80 to 65% B; 5 – 7 min from 65 to 20% B; 7 – 9 min constant at 20% B; 9 – 10 min from 20 to 80% B; 10 – 12 min constant at 80% B. An Agilent 6495 ion funnel mass spectrometer was used in positive mode with an electrospray ionization source and the following conditions: ESI spray voltage 2000 V, sheath gas 250°C at 12 l/min, nebulizer pressure 50 psig and drying gas 100°C at 11 l/min. Compounds were identified based on their mass transition and retention time compared to standards. Chromatograms were integrated using MassHunter software (Agilent, Santa Clara, CA, USA). Absolute concentrations of Asp-^{15}N and Asp were calculated based on an external calibration curve of Asp-^{13}C prepared in sample matrix after confirming that the uniformly labelled analyte cannot be detected in the matrix prior to standard addition, and after confirming that the correlation between the signal intensity and concentration of the ^{13}C-labelled analyte equals the correlation of the unlabeled as well as the ^{15}N-labelled amino acid by standard addition. Quantification via an isotopically labelled external standard was required, as the unlabeled analyte that was found in the matrix in high abundance interfered with the measurement.

SDS-PAGE and immunoblot analysis

SDS-PAGE and immunoblot analysis were performed as described in (4, 5). 15 µg total leaf was loaded for SDS-PAGE. For immunoblot analysis monoclonal conjugated horseradish peroxidase anti-HA antibody (Miltenyi Biotech) was used for the detection of AGAT and ISR. Monoclonal conjugated horseradish peroxidase anti-His antibody (Miltenyi Biotech) was used for the detection of BHAA and BHAD.

Quantitative western blots

Total protein was extracted from four-weeks-old mature rosettes grown under ambient CO_2 with TCA/acetone as described by (6). For quantification commercial standards of Rubisco large subunit (RbcL, Agrisera) and the Rieske FeS protein of cytochrome b_6f complex, PetC (Agrisera) as well as RbcL and PetC antibodies (Agrisera) were used in defined amounts. Protein concentration for quantification was assessed by dilution series using 50 µg, 25 µg 12.5 µg, 6.25 µg and 3.125 µg total protein. 6.25 ug total protein was loaded per lane and the signal intensity was determined with the Image Studio Lite software (Version 5.2.5, LI-COR). Signal intensity was normalized to a 0.25 pmol protein standard.

Plant phenotyping

For fresh weight and dry weight analysis, 12-days-old seedlings were harvested. Seedlings were dried for four days at 65°C before dry weight analysis. Rosette area and rosette diameter were quantified on photographed pots via Fiji.

Chlorophyll fluorescence measurements

Chlorophyll photochemical efficiency of photosystem II in dark-adapted leaves (F_v/F_m) (7) was measured on 12-days-old seedlings using an imaging chlorophyll fluorometer (Imaging PAM, Walz). Upon dark adaptation for 20 min, seedlings were exposed to a pulsed, blue probe beam and a saturating light flash to measure F_v/F_m values.

Free ammonium quantification

Free ammonium was quantified in plant tissue using a colorimetric assay as described previously (8).

Metabolite profiling by GC/MS Q-TOF

Frozen material was ground using precooled mortar and pestle. Grinded material was aliquoted under continuous liquid nitrogen exposure to avoid sample thawing. Extraction mix, containing water:methanol:chloroform (ratio 1:2.5:1) and 5 µM ribitol as internal standard, was added to frozen material. Samples were vortexed for 20 seconds, rotated for 6 min at 4°C and centrifuged for 2 min at 20,000 x g at room temperature. The supernatant was transferred to a new tube and stored at -80°C before further processing. For metabolite profiling by gas-chromatography time of flight mass spectrometry (GC/MS Q-TOF), 50 µl of extract was dried using a speed vacuum concentrator. Dried samples were placed in the Gerstel MPS 2 XL autosampler for automatic sample derivatization using methoxyamine hydrochloride and N-Methyl-N-(trimethylsilyl) trifluoroacetamide before injection. The GC-MS device is a 7200 accurate mass Q-TOF GC/MS (Agilent). For relative quantification metabolite peak areas are normalized to the internal extraction standard and the material fresh weight.

Metabolite profiling by IC/MS and data analysis

The extraction mix contained 5 µM thio-ATP and itaconate as internal standards. For IC-MS a combination of a Dionex ICS-6000 HPIC and a high field Q Exactive Plus quadrupole-Orbitrap mass spectrometer (Thermo Fisher Scientific) was used. The chromatographic method and mass spectrometry settings were adopted from (9) with minor adjustments. The dried sample was reconstituted in 150 µl deionized water and 5 µl were injected via a Dionex AS-AP autosampler in push partial mode with a 10 µL sample loop. The full scan (60-800 m/z) was conducted with a resolution of 140.000 and an automatic gain control target of 1e6 ions with a maximum injection time of 500 ms. The Top5 ddMS2 experiment was carried out with a resolution of 17.500 and an AGC target of 1e5 and a maximum IT of 50 ms. The stepped collision energy was used with the steps 15, 25 and 35). Untargeted data analysis was conducted with Compound Discoverer (version 3.1, Thermo Fisher Scientific) using the “untargeted Metabolomics workflow”. Retention time

alignment was performed in a window of 2 min within 5 ppm mass accuracy. Elemental compositions were predicted based on accurate mass and chemical background was substracted by using blank extraction samples. Chemical sum compositions were calculated based on the accurate mass (3 ppm mass accuracy) on MS1 level and searched against Chemspider (http://www.chemspider.com) with ranking by the mzLogic algorithm. Furthermore, the MS2 fragment spectra were matched against the mzCloud library and an in-house build MS2 spectral library. QC-based batch normalization was performed with QC sample injection every 5 samples during the sequence.

Fig. S1. BHAC implementation in *Arabidopsis thaliana* wild type *Col-0* and *ggt1-1* mutant background. A) Schematic representation of the multigene T-DNA construct for BHAC pathway implementation. Aspartate:glyoxylate aminotransferase (AGAT), β-hydroxyaspartate aldolase (BHAA), β-hydroxyaspartate dehydratase (BHAD), iminosuccinate reductase (ISR). Kanamycin resistance (KanR). B and C) Genotyping of transgenic homozygous T3 BHAC plants in wild type *Col-0* (B) and *ggt1-1* mutant background (C). D and E) Immunoblot analysis of BHAC enzyme expression in BHAC plants. BHAA and BHAD were detected with an anti-HIS-HRP antibody (D) and AGAT and ISR with an anti-HA-HRP antibody (E). Arabidopsis Rubisco large subunit (RbcL) served as loading control and was visualized by ponceau staining. 15 μg total leaf protein of four-weeks-old air-grown plants was loaded per lane. PageRuler Plus prestained protein ladder was used (L).

Fig. S2. β-hydroxyaspartate detection by GC-MS QTOF. A) Deconvoluted mass spectrum of *erythro*-β-hydroxyaspartate. B) Extracted ion-chromatogram of *erythro*-β-hydroxyaspartate specific masses in wild type *Col-0* extract spiked with 20 µM analytical standard of *D-erythro*-β-hydroxyaspartate. C) Deconvoluted mass spectrum of *threo*-β-hydroxyaspartate. D) Extracted ion-chromatogram of *threo*-β-hydroxyaspartate specific masses in wild type *Col-0* extract spiked with 20 µM analytical standard of *DL-threo*-β-hydroxyaspartate.

Fig. S3. *In planta* β-hydroxyaspartate formation. A and B) Representative extracted ion chromatograms of the β-hydroxyaspartate specific masses. *In vivo* formation of both diastereomers, *erythro*-β-hydroxyaspartate (A) and *threo*-β-hydroxyaspartate (B) is shown in one T-DNA line per background genotype.

Fig. S4. Metabolome profile of BHAC plants. Metabolite profiles were generated using green tissue of 14-days-old seedlings grown either in CO_2 enriched air (3000 ppm CO_2, HC), ambient air (400 ppm CO_2, AC) or shifted from HC to AC three days prior to harvest (Shift). Each box-whisker plot represents the 25th and 75th percentiles and whiskers the 10th and 90th percentile. Median is indicated as crossbar. One-way ANOVA with a post-hoc Tukey HSD test was used for statistical analysis. Different letters indicate significant differences between genotypes at $p<0.05$. n = 4.

Fig. S5. Peroxisomal aspartate:glyoxylate aminotransferase restores canonical photorespiration in the *ggt1-1* mutant. The *ggt1-1* mutant was complemented by expression of aspartate:glyoxylate aminotransferase under the chlorophyll A/B binding protein 1 promoter (*ggt1-1::AGAT*). Numbers indicate independent T-DNA lines. A) Representative images of seedlings for F_v/F_m measurements using 12-days-old seedlings grown at ambient air (400 ppm CO_2, AC) or in CO_2 enriched air (3000 ppm CO_2, HC). Scalebar = 0.5 cm. B) Quantification of F_v/F_m values of plants grown at AC (top) or HC (bottom). Student's *t*-test against wild type *Col-0* was used for statistical analysis. p < 0.05 = *, < 0.01 = **, < 0.001 = ***. n >25 per genotype per condition. C) *In vitro* glutamate:glyoxylate (GGT) and aspartate:glyoxylate (AGAT) activity. Activity was measured in three biological replicates in technical triplicates using mature rosette leaves of four-weeks-old air-grown plants. D) Relative metabolite levels in *ggt1-1::AGAT* complementation lines grown in air. Student's *t*-test against wild type *Col-0* was used for statistical analysis. Asterisks indicate significance after multiple testing correction using Benjamini-Hochberg. p < 0.05 = *, < 0.01 = **, < 0.001 = ***. n = 4. E) Images of plants grown in ambient air (400 ppm CO_2) at 21 days (top) and 28 days (bottom) after transfer to light. F) Rosette area (left) and rosette diameter (right) of ambient air grown plants. Student's *t*-test against wild type *Col-0* was used for statistical analysis. p < 0.05 = *, p < 0.01 = **, p < 0.001 = ***. Colored asterisks represent the significance for the respective genotype. n = 3. Shown are mean ± SD.

Fig. S6. Phenotyping of BHAC plants at seedling stage. A) Representative images of BHAC plants for F_v/F_m measurements using 12-days-old seedling grown at 400 ppm CO_2 (AC) 3000 ppm CO_2 (HC) or shifted from 3000 ppm CO_2 to 400 ppm CO_2 three days prior harvest (Shift). Scalebar = 0.5 cm. B) to D) Quantification of F_v/F_m values of plants grown at AC (B), HC (C) or shifted (D). Student's *t*-test against wild type *Col-0* was used for statistical analysis. $p < 0.05$ = *, < 0.01 = **, < 0.001 = ***. n >25 per genotype per condition. E to H) Seedling fresh weight (E and F) and dry weight (G and H) of 12-days-old seedling grown at AC (E and G) or HC (F and H). Student's *t*-test against wild type *Col-0* was used for statistical analysis. $p < 0.05$ = *, < 0.01 = **, < 0.001 = ***. Shown are mean ± SD. n = 4.

Fig. S7. Growth of BHAC containing plants in wild type *Col-0* (A) or *ggt1-1* mutant (B) background. Rosette area and rosette diameter were quantified over time for plants grown under AC (top) or HC (bottom). Student's *t*-test against background genotype was used for statistical analysis. Shown wild type in (B) is same as in (A) and added for comparative reasons. Colored asterisks represent the significance for the respective genotype. $p < 0.05$ = *, $p < 0.01$ =**, < 0.001 = ***. n = 5.

Fig. S8. Relative levels of glyoxylate, adenine nucleotides and phosphorylated sugars in BHAC plants grown in ambient air. Each box-whisker plot represents the 25th and 75th percentiles and whiskers the 10th and 90th percentile. Median is indicated as crossbar. One-way ANOVA with a post-hoc Tukey HSD test was used for statistical analysis. Different letters indicate significant differences between genotypes at $p<0.05$. $n \geq 3$.

Fig. S9. A/C_i curves and light response curves of BHAC plants. A & B) CO_2 assimilation based on intracellular CO_2 concentration (C_i) for BHAC plants in wild type *Col-0* (A) and *ggt1-1* background (B). C & D) CO_2 assimilation based on photosynthetic active radiation (PAR). Shown wild type *Col-0* in the bottom panels is the same as the in A) and C) respectively and added for comparative reasons. Shown are mean ± SD. n = 4 per genotype.

Fig. S10. Quantitative Western-Blots. A) Representative western blot used for quantification of rubisco large subunit (RbcL) and Rieske-Fe (PetC). B) Relative signal intensity. Signal intensity for each genotype was normalized to a 0.25 pmol RbcL and PetC protein standard respectively. One-way ANOVA with a post-hoc Tukey HSD test was used for statistical analysis. Different letters indicate significant differences between genotypes at $p<0.05$. Shown are mean ± SD, n = 3.

Fig. S11. A BHAC-derived synthetic C4 cycle. Schematic representation of plant photorespiration (PR), BHAC and potential routes for a synthetic C4 cycle between mesophyll (MS) and bundle sheath cells (BS). These include the decarboxylation of malate or malate catabolism and acetyl-CoA decarboxylation and glycine decarboxylation (Gly decar.) or regeneration (Gly reg.). Abbreviations: Aspartate:glyoxylate aminotransferase (AGAT), β-hydroxyaspartate aldolase (BHAA), β-hydroxyaspartate dehydratase (BHAD), iminosuccinate reductase (ISR), glutamate:glyoxylate aminotransferase (GGT1), ribulose-1,5-bisphosphate (RuBP), plastidial glycolate/glycerate transporter 1 (PLGG1), bile-acid sodium symporter 6 (BASS6), malate synthase (MSyn), aminotransferase (AT), malic enzyme (ME), pyruvate dehydrogenase (PDH).

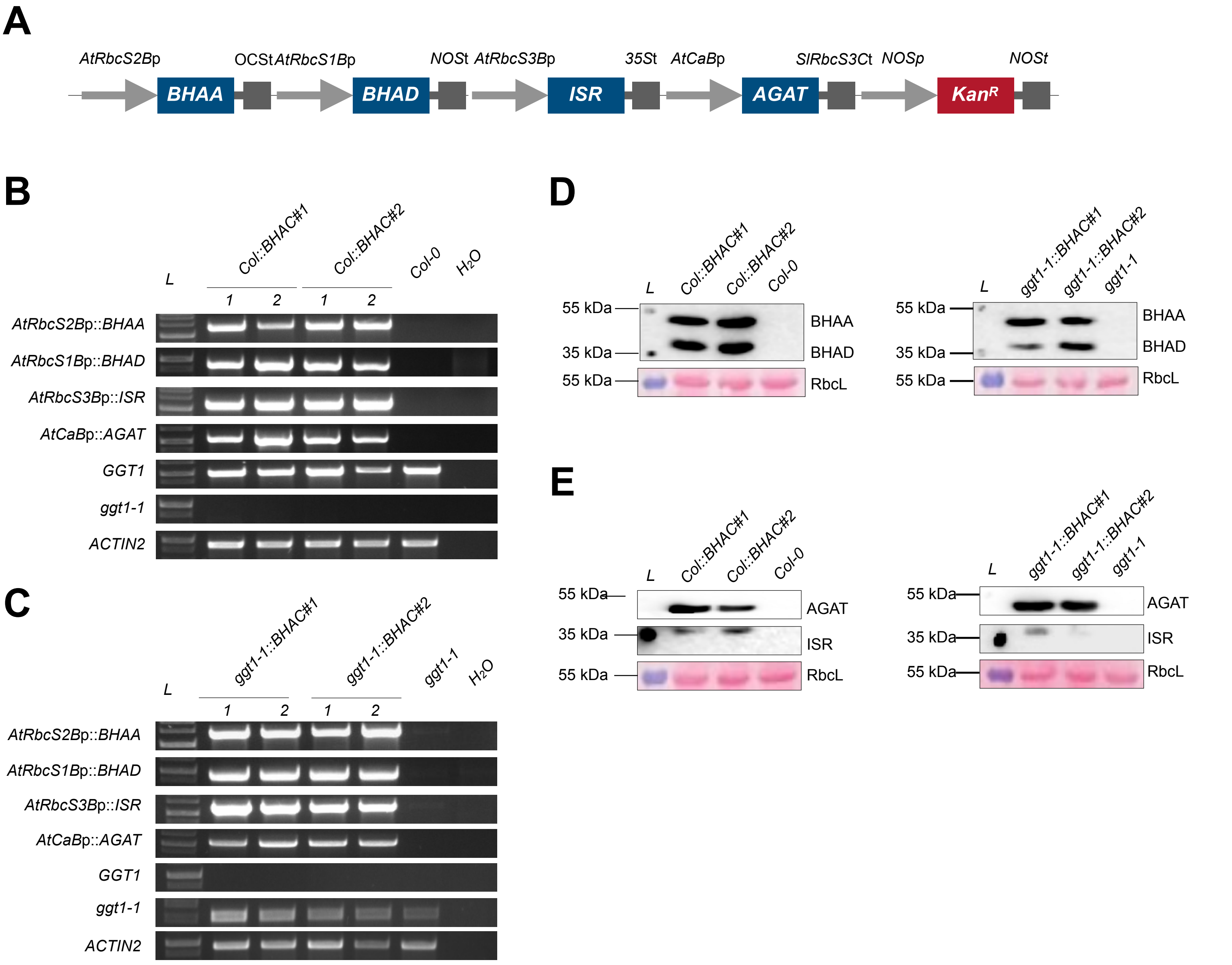

Fig. S1. BHAC implementation in *Arabidopsis thaliana* wild type *Col-0* and *ggt1-1* mutant background. A) Schematic representation of the multigene T-DNA construct for BHAC pathway implementation. Aspartate:glyoxylate aminotransferase (AGAT), β-hydroxyaspartate aldolase (BHAA), β-hydroxyaspartate dehydratase (BHAD), iminosuccinate reductase (ISR). Kanamycin resistance (KanR). B and C) Genotyping of transgenic homozygous T3 BHAC plants in wild type *Col-0* (B) and *ggt1-1* mutant background (C). D and E) Immunoblot analysis of BHAC enzyme expression in BHAC plants. BHAA and BHAD were detected with an anti-HIS-HRP antibody (D) and AGAT and ISR with an anti-HA-HRP antibody (E). Arabidopsis Rubisco large subunit (RbcL) served as loading control and was visualized by ponceau staining. 15 µg total leaf protein of four-weeks-old air-grown plants was loaded per lane. PageRuler Plus prestained protein ladder was used (L).

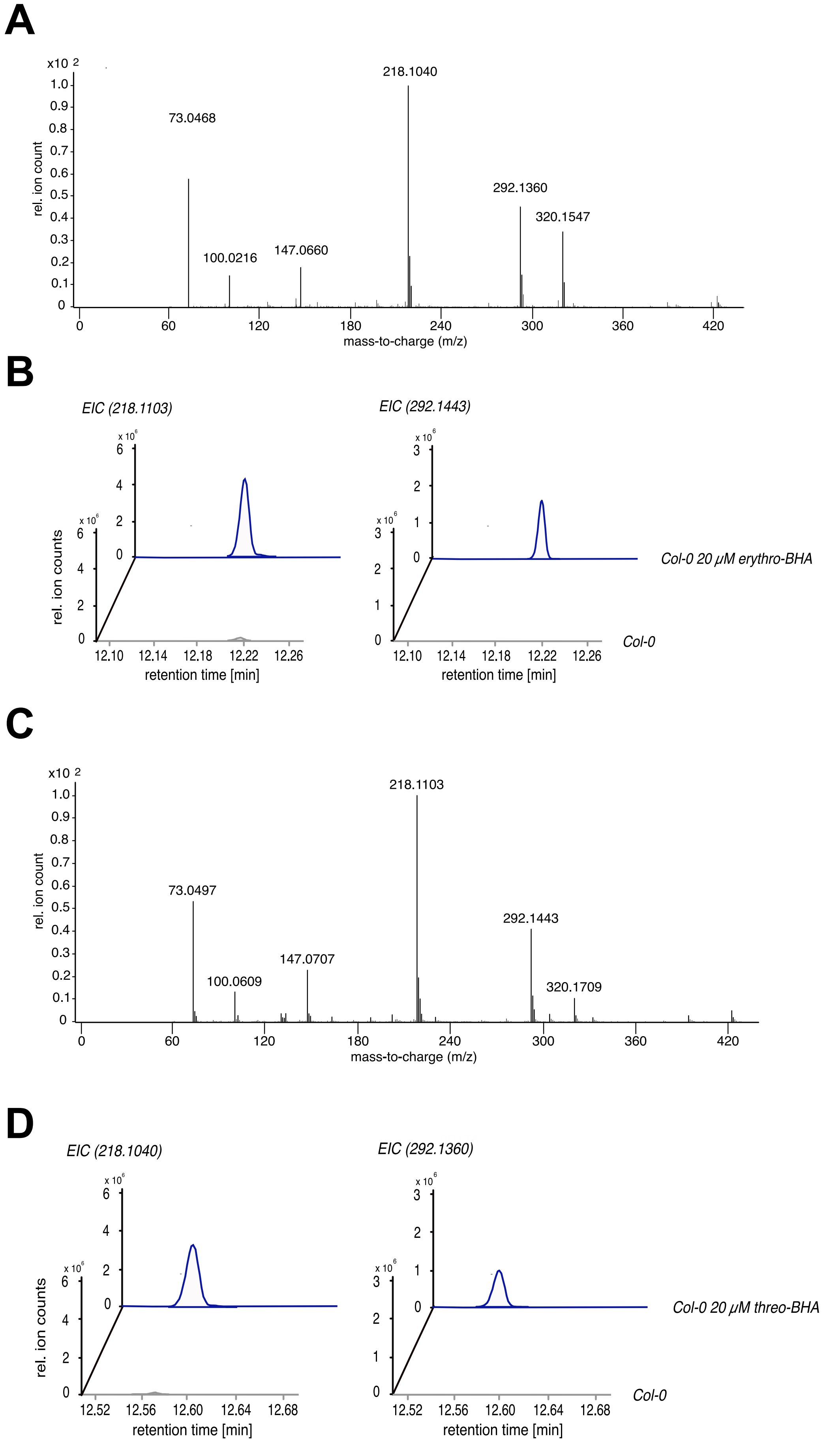

Fig. S2. β-hydroxyaspartate detection by GC-MS QTOF. A) Deconvoluted mass spectrum of *erythro*-β-hydroxyaspartate. B) Extracted ion-chromatogram of *erythro*-β-hydroxyaspartate specific masses in wild type *Col-0* extract spiked with 20 µM analytical standard of *D-erythro*-β-hydroxyaspartate. C) Deconvoluted mass spectrum of *threo*-β-hydroxyaspartate. D) Extracted ion-chromatogram of *threo*-β-hydroxyaspartate specific masses in wild type *Col-0* extract spiked with 20 µM analytical standard of *DL-threo*-β-hydroxyaspartate.

A

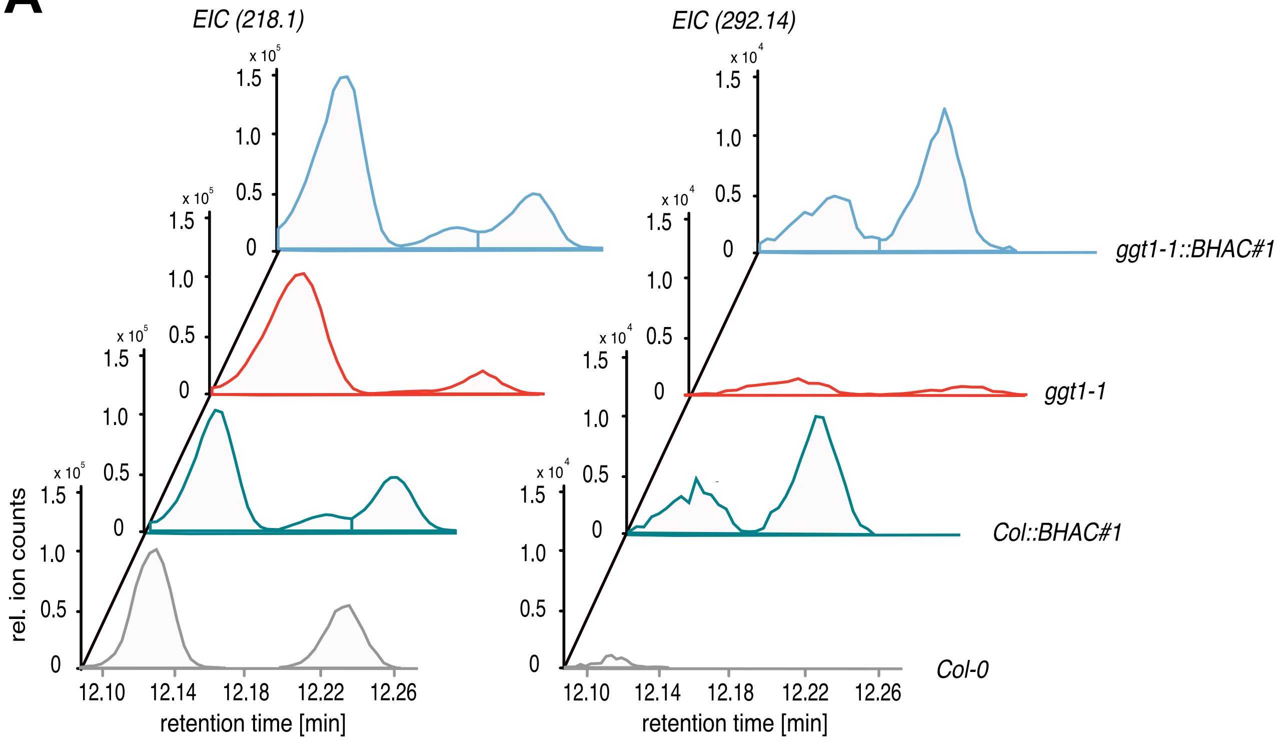

B

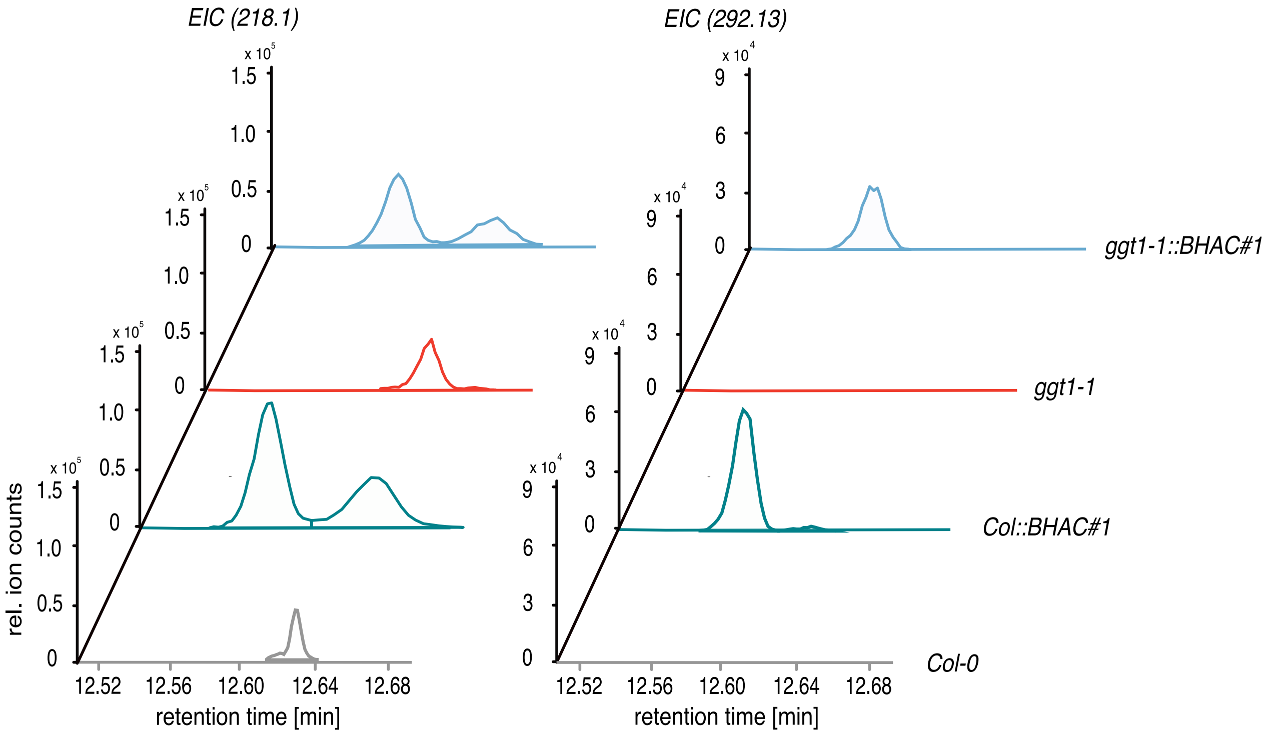

Fig. S3. *In planta* β-hydroxyaspartate formation. A and B) Representative extracted ion chromatograms of the β-hydroxyaspartate specific masses. *In vivo* formation of both diastereomers, *erythro*-β-hydroxyaspartate (A) and *threo*-β-hydroxyaspartate (B) is shown in one T-DNA line per background genotype.

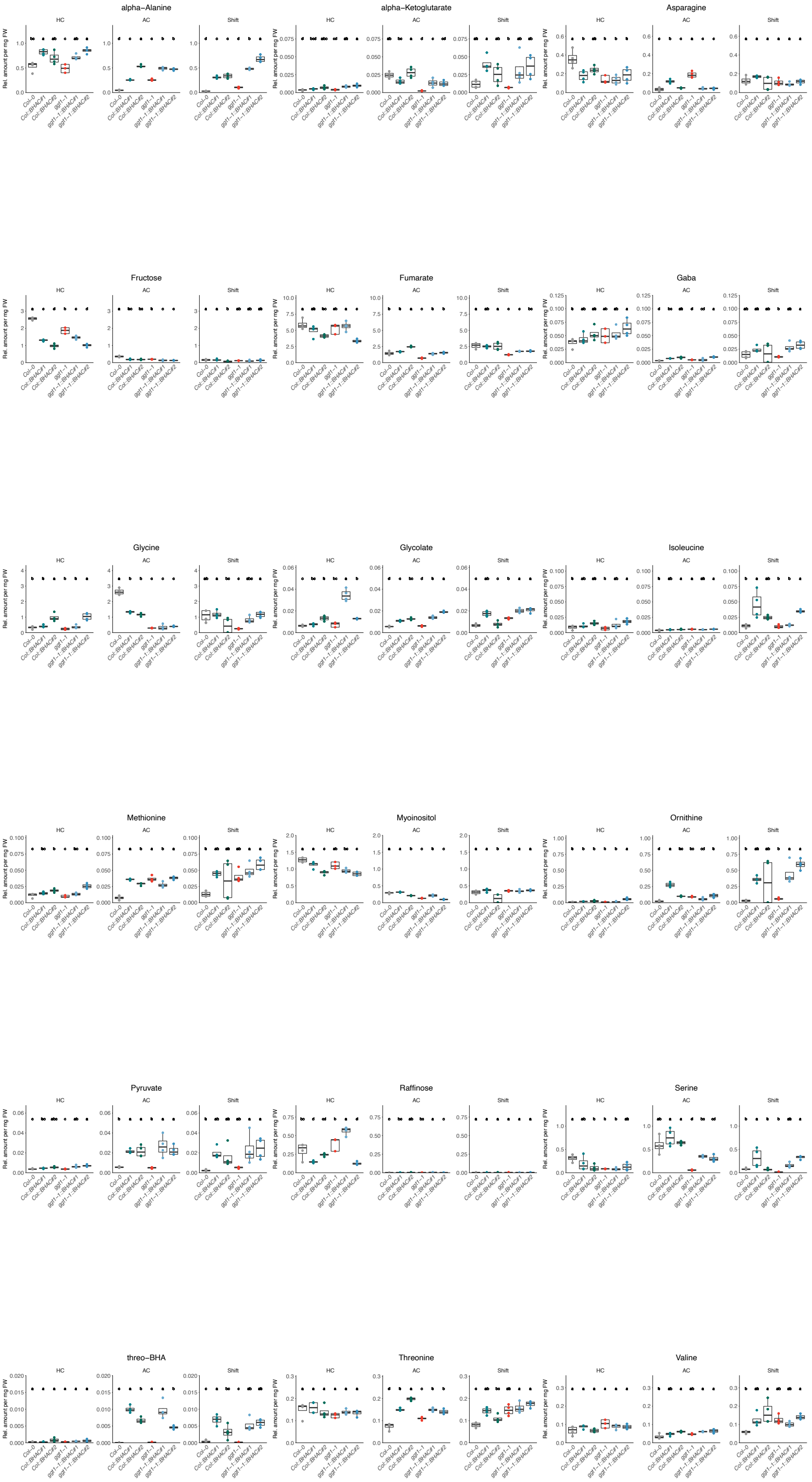

Fig. S4. Metabolome profile of BHAC plants. Metabolite profiles were generated using green tissue of 14-days-old seedlings grown either in CO_2 enriched air (3000 ppm CO_2, HC), ambient air (400 ppm CO_2, AC) or shifted from HC to AC three days prior to harvest (Shift). Each box-whisker plot represents the 25th and 75th percentiles and whiskers the 10th and 90th percentile. Median is indicated as crossbar. One-way ANOVA with a post-hoc Tukey HSD test was used for statistical analysis. Different letters indicate significant differences between genotypes at $p<0.05$. n = 4.

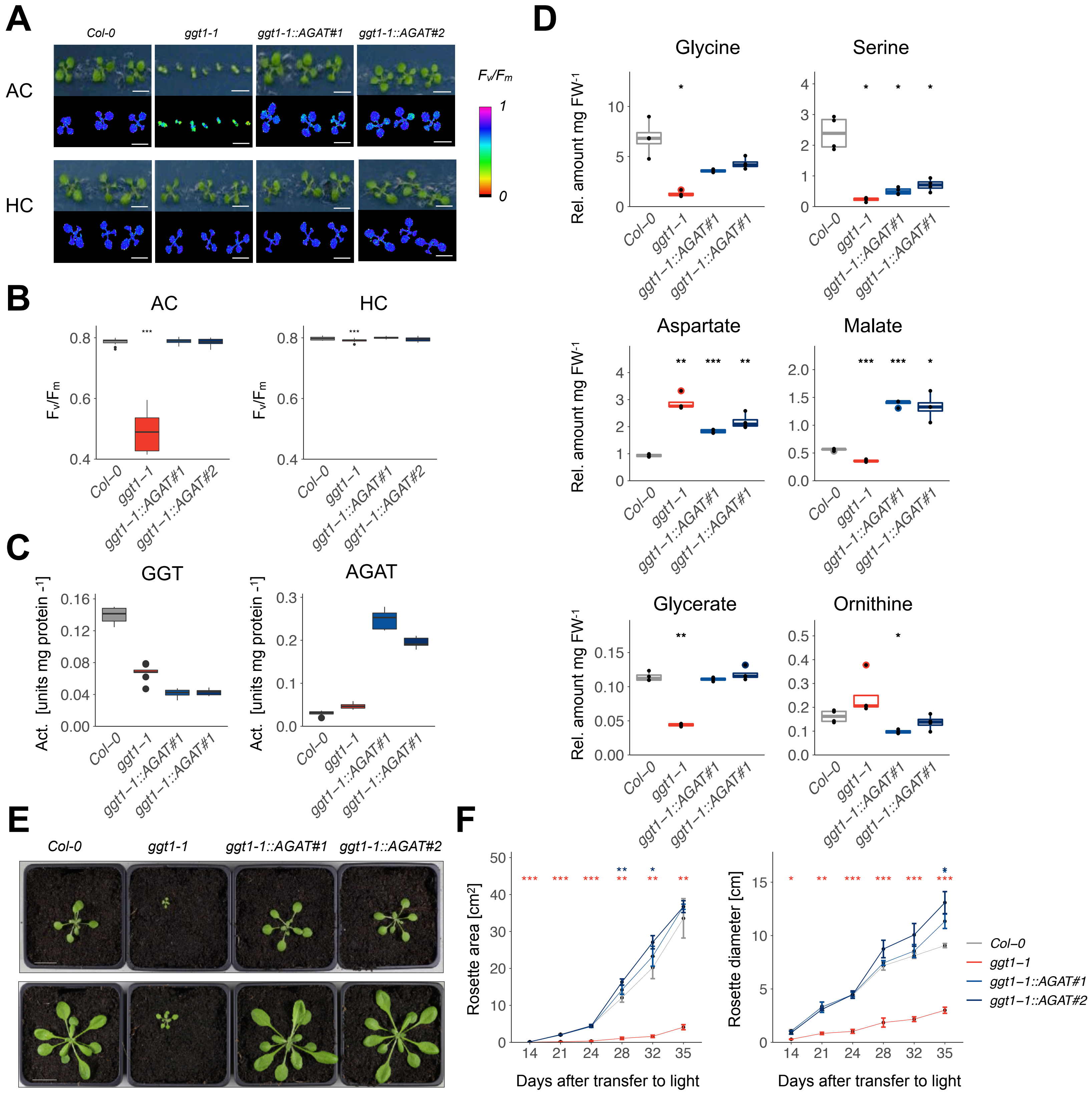

Fig. S5. Peroxisomal aspartate:glyoxylate aminotransferase restores canonical photorespiration in the *ggt1-1* mutant. The *ggt1-1* mutant was complemented by expression of aspartate:glyoxylate aminotransferase under the chlorophyll A/B binding protein 1 promoter (*ggt1-1::AGAT*). Numbers indicate independent T-DNA lines. A) Representative images of seedlings for F_v/F_m measurements using 12-days-old seedlings grown at ambient air (400 ppm CO_2, AC) or in CO_2 enriched air (3000 ppm CO_2, HC). Scalebar = 0.5 cm. B) Quantification of F_v/F_m values of plants grown at AC (top) or HC (bottom). Student's *t*-test against wild type *Col-0* was used for statistical analysis. p < 0.05 = *, < 0.01 = **, < 0.001 = ***. n >25 per genotype per condition. C) *In vitro* glutamate:glyoxylate (GGT) and aspartate:glyoxylate (AGAT) activity. Activity was measured in three biological replicates in technical triplicates using mature rosette leaves of four-weeks-old air-grown plants. D) Relative metabolite levels in *ggt1-1::AGAT* complementation lines grown in air. Student's *t*-test against wild type *Col-0* was used for statistical analysis. Asterisks indicate significance after multiple testing correction using Benjamini-Hochberg. p < 0.05 = *, < 0.01 = **, < 0.001 = ***. n = 4. E) Images of plants grown in ambient air (400 ppm CO_2) at 21 days (top) and 28 days (bottom) after transfer to light. F) Rosette area (left) and rosette diameter (right) of ambient air grown plants. Student's *t*-test against wild type *Col-0* was used for statistical analysis. p < 0.05 = *, p < 0.01 = **, p < 0.001 = ***. Colored asterisks represent the significance for the respective genotype. n = 3. Shown are mean ± SD.

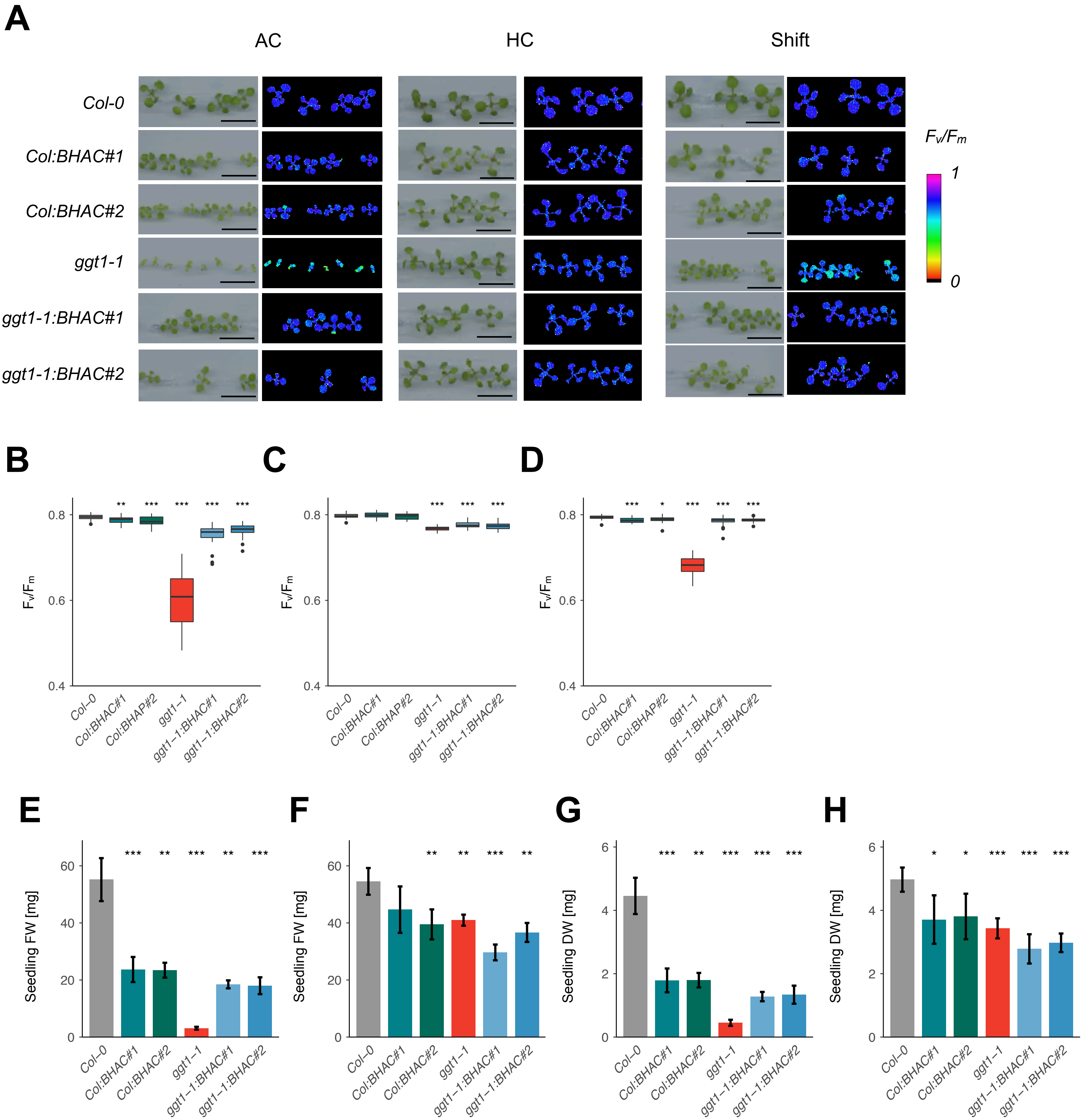

Fig. S6. Phenotyping of BHAC plants at seedling stage. A) Representative images of BHAC plants for F_v/F_m measurements using 12-days-old seedling grown at 400 ppm CO_2 (AC) 3000 ppm CO_2 (HC) or shifted from 3000 ppm CO_2 to 400 ppm CO_2 three days prior harvest (Shift). Scalebar = 0.5 cm. B) to D) Quantification of F_v/F_m values of plants grown at AC (B), HC (C) or shifted (D). Student's *t*-test against wild type *Col-0* was used for statistical analysis. $p < 0.05$ = *, < 0.01 = **, < 0.001 = ***. n >25 per genotype per condition. E to H) Seedling fresh weight (E and F) and dry weight (G and H) of 12-days-old seedling grown at AC (E and G) or HC (F and H). Student's *t*-test against wild type *Col-0* was used for statistical analysis. $p < 0.05$ = *, < 0.01 = **, < 0.001 = ***. Shown are mean ± SD. n = 4.

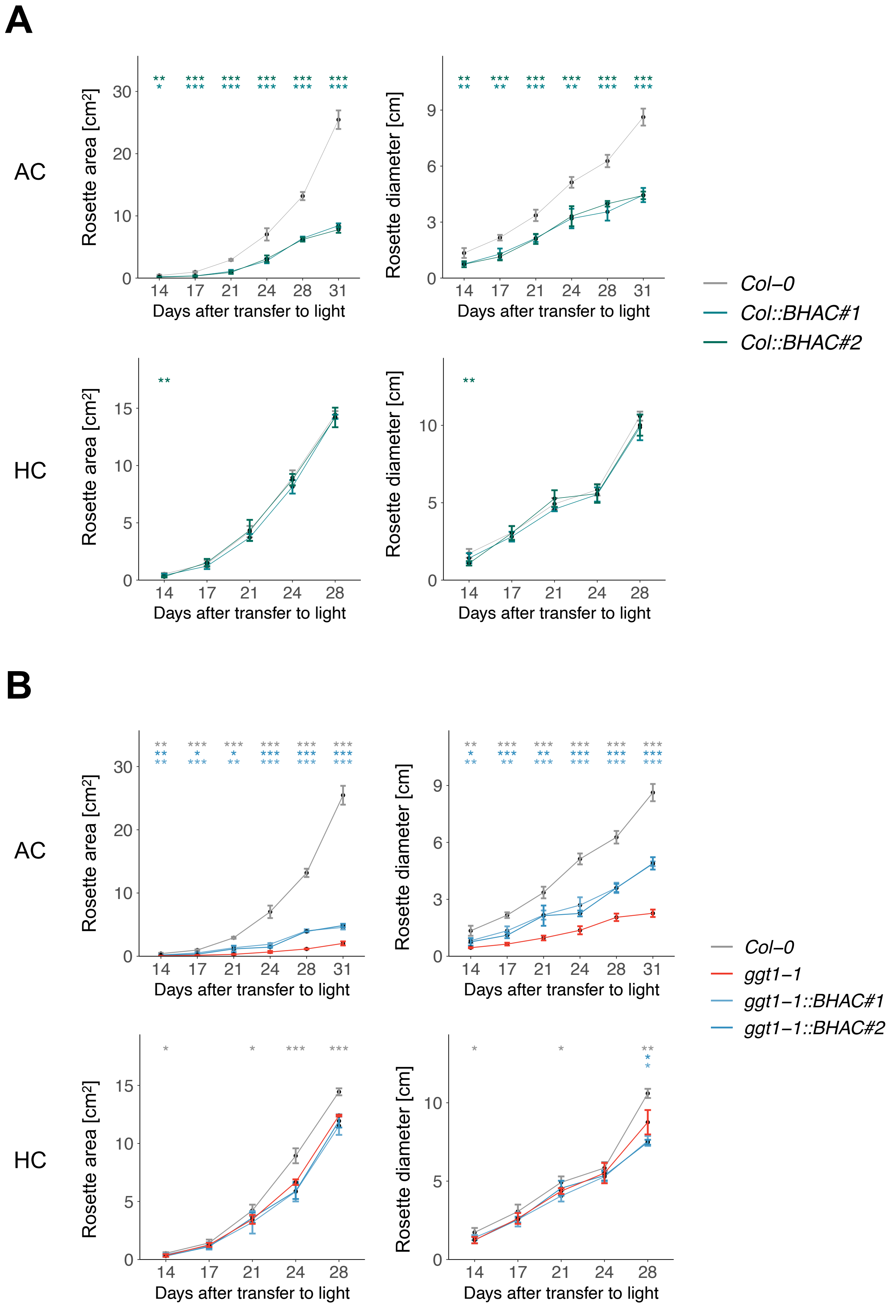

Fig. S7. Growth of BHAC containing plants in wild type *Col-0* (A) or *ggt1-1* mutant (B) background. Rosette area and rosette diameter were quantified over time for plants grown under AC (top) or HC (bottom). Student's *t*-test against background genotype was used for statistical analysis. Shown wild type in (B) is same as in (A) and added for comparative reasons. Colored asterisks represent the significance for the respective genotype. p< 0.05 = *, p < 0.01 =**, < 0.001 = ***. n = 5.

A

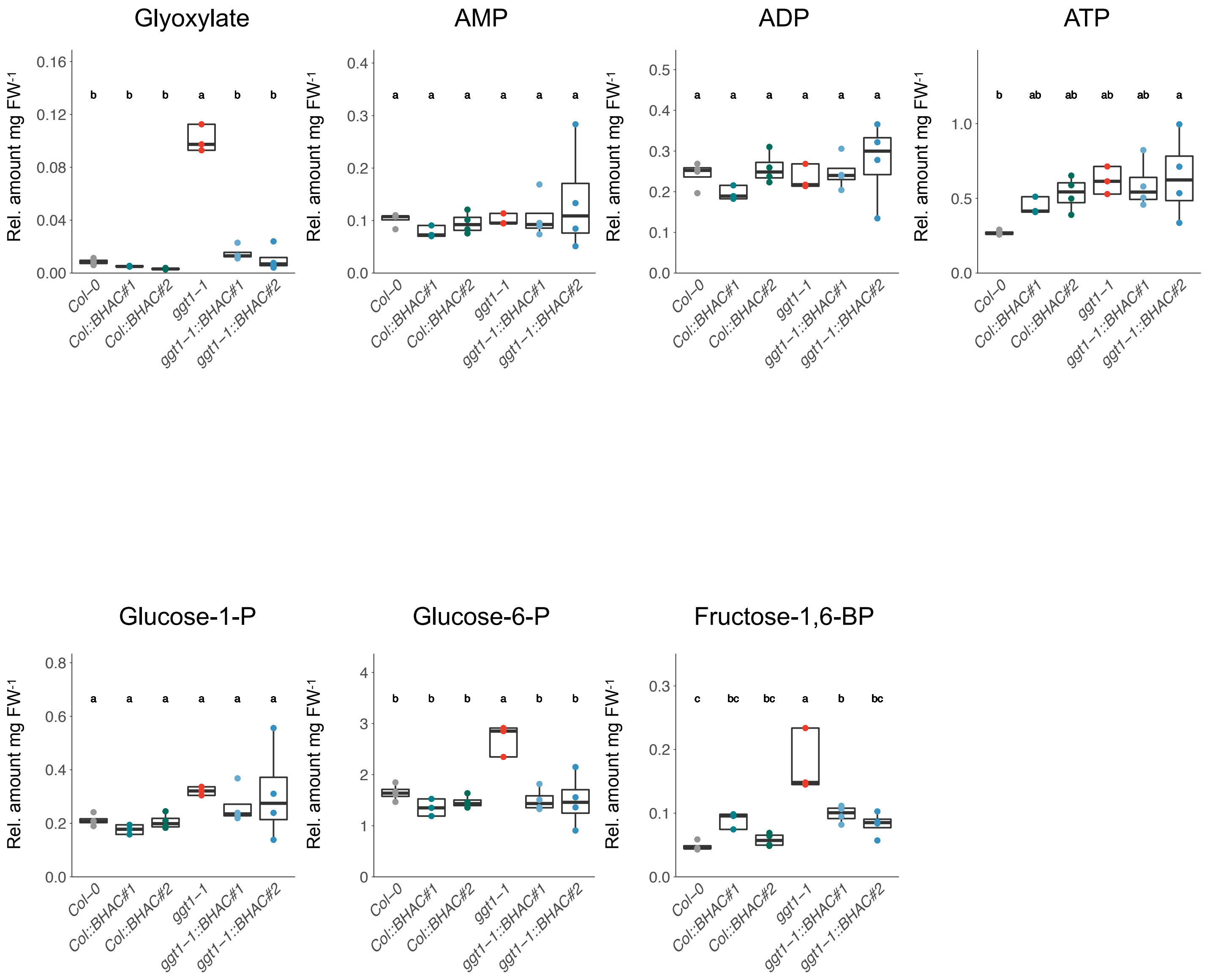

Fig. S8. Relative levels of glyoxylate, adenine nucleotides and phosphorylated sugars in BHAC plants grown in ambient air. Each box-whisker plot represents the 25th and 75th percentiles and whiskers the 10th and 90th percentile. Median is indicated as crossbar. One-way ANOVA with a post-hoc Tukey HSD test was used for statistical analysis. Different letters indicate significant differences between genotypes at $p<0.05$. $n \geq 3$.

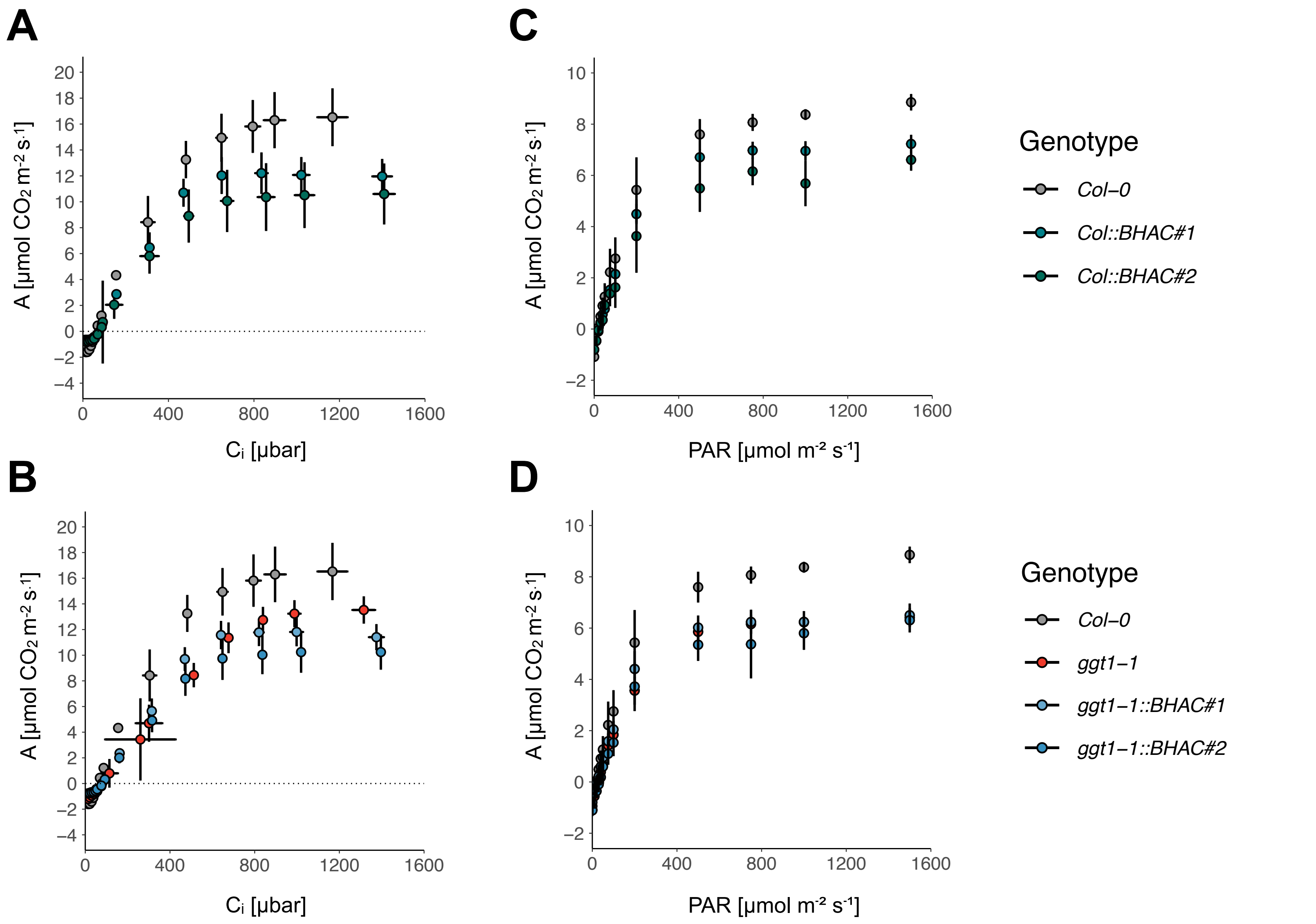

Fig. S9. A/C_i curves and light response curves of BHAC plants. A & B) CO_2 assimilation based on intracellular CO_2 concentration (C_i) for BHAC plants in wild type *Col-0* (A) and *ggt1-1* background (B). C & D) CO_2 assimilation based on photosynthetic active radiation (PAR). Shown wild type *Col-0* in the bottom panels is the same as the in A) and C) respectively and added for comparative reasons. Shown are mean ± SD. n = 4 per genotype.

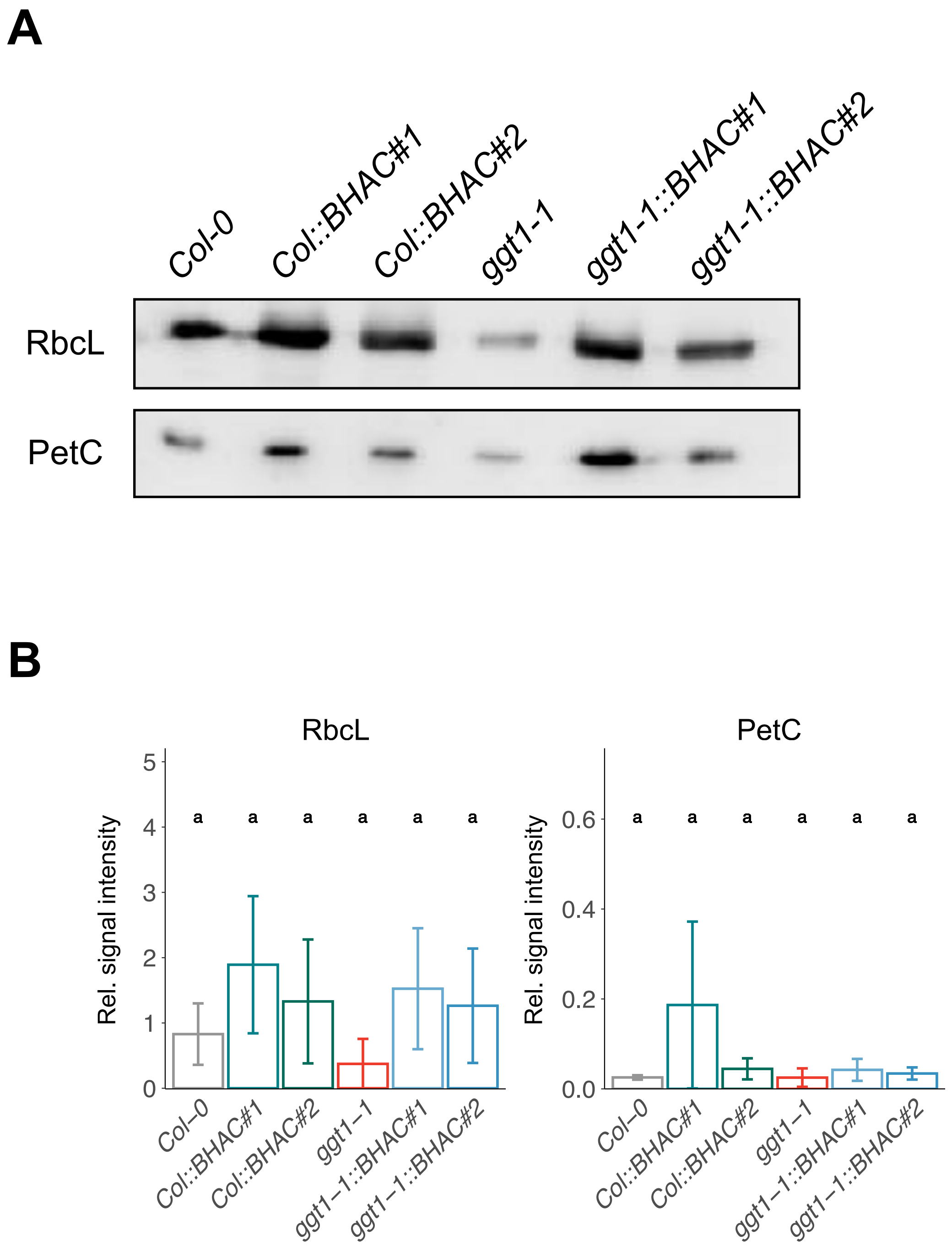

Fig. S10. Quantitative Western-Blots. A) Representative western blot used for quantification of rubisco large subunit (RbcL) and Rieske-Fe (PetC). B) Relative signal intensity. Signal intensity for each genotype was normalized to a 0.25 pmol RbcL and PetC protein standard respectively. One-way ANOVA with a post-hoc Tukey HSD test was used for statistical analysis. Different letters indicate significant differences between genotypes at $p<0.05$. Shown are mean ± SD, n = 3.

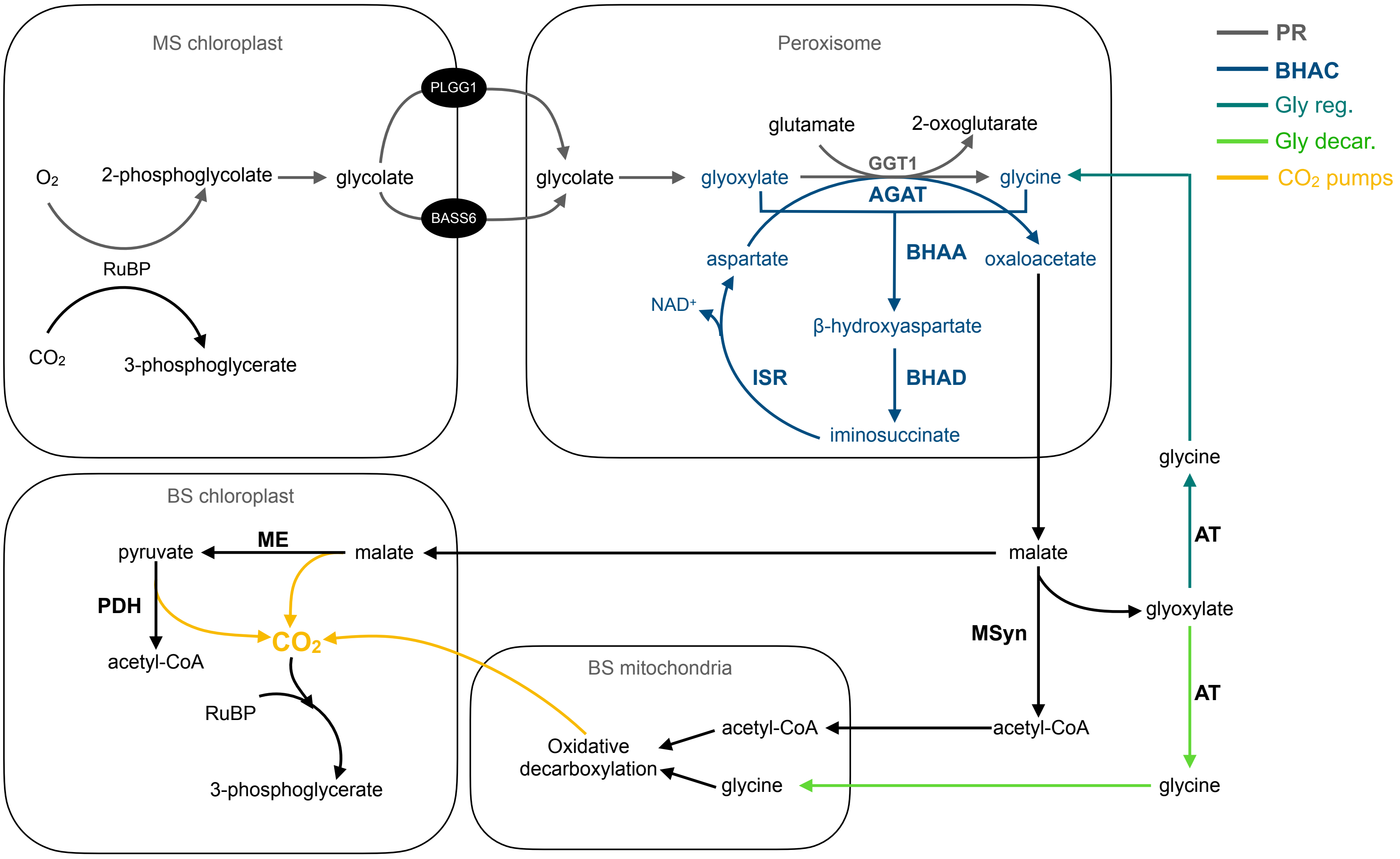

Fig. S11. A BHAC-derived synthetic C4 cycle. Schematic representation of plant photorespiration (PR), BHAC and potential routes for a synthetic C4 cycle between mesophyll (MS) and bundle sheath cells (BS). These include the decarboxylation of malate or malate catabolism and acetyl-CoA decarboxylation and glycine decarboxylation (Gly decar.) or regeneration (Gly reg.). Abbreviations: Aspartate:glyoxylate aminotransferase (AGAT), β-hydroxyaspartate aldolase (BHAA), β-hydroxyaspartate dehydratase (BHAD), iminosuccinate reductase (ISR), glutamate:glyoxylate aminotransferase (GGT1), ribulose-1,5-bisphosphate (RuBP), plastidial glycolate/glycerate transporter 1 (PLGG1), bile-acid sodium symporter 6 (BASS6), malate synthase (MSyn), aminotransferase (AT), malic enzyme (ME), pyruvate dehydrogenase (PDH).

Table S1. List of primers used in this study. Gene specific nucleotides indicated in caps. Abbreviations: GGT1 (glutamate:glyoxylate aminotransferase 1, At1g23310).

Primer sequence	Purpose	Reference
fwd: 5'-CCTTGCCCTTGGCTCTAGAACC-3'<br>rev: 5'-GTCATACCTAAACCGCCTGAAGTC-3'	*Genotyping GGT1*	This Study
fwd: 5'-TAACTCTCCCCACTCTTTGCC-3'<br>rev: 5'-ATATTGACCATCATACTCATTGC-3'	T-DNA primer *ggt1-1*	

Table S2. List of constructs used in this study. Vector backbones, promoters and terminators are described in (10). Abbreviations: aspartate:glyoxylate aminotransferase (AGAT), β-hydroxyaspartate aldolase (BHAA), β-hydroxyaspartate dehydratase (BHAD), iminosuccinate reductase (ISR). PTS1: peroxisomal target signal 1, PTS2: peroxisomal target signal 2. BHAC: β-hydroxyaspartate cycle. *Arabidopsis thaliana* (*At*), *Solanum lycopersicum* (*Sl*), *Agrobacterium tumefaciens* (*Atu*).

**Purpose**	**Vector**	**Insert**	**Comment**
Localization AGAT	pICH86966	*UBQ10*p::mCherry-AGAT$_{PTS1}$::*Sl*Rbsc3cT	PTS1: serine-lysine-leucine
Localization BHAA	pICH86966	*UBQ10*p::$_{PTS2}$BHAA-mCherry::*Sl*Rbsc3cT	PTS2 from At citrate synthase (At2g42790)
Localization BHAD	pICH86966	*UBQ10*p::mCherry-BHAD$_{PTS1}$::*SlRbsc3c*T	PTS1: serine-lysine-leucine
Localization ISR	pICH86966	*UBQ10*p::eGFP-ISR$_{PTS1}$::*SlRbsc3c*T	PTS1: serine-lysine-leucine
ggt1-1 complementation with AGAT	pICH86966	AtCABp::AGAT$_{PTS1}$:: *Sl*Rbsc3cT	Kanamycin resistance for plants
BHAC T-DNA construct for implementation in plants	pICH75322	Pos. 1: *At*RbcS2Bp::$_{PTS2}$BHAA::*Atu*OcsT<br>Pos. 2: *At*RbcS1Bp::BHAD$_{PTS1}$::AtuNosT<br>Pos. 3: *At*RbcS3Bp::ISR$_{PTS1}$::35sT<br>Pos. 4: *At*CABp::AGAT$_{PTS1}$:: *Sl*Rbsc3cT<br>Pos. 5: *Atu*Nosp::NptII::*Atu*OcsT	Kanamycin resistance for plants

SI References

1. C. Koncz, J. Schell, The promoter of TL-DNA gene 5 controls the tissue-specific expression of chimaeric genes carried by a novel type of Agrobacterium binary vector. *Mol Gen Genet* **204**, 383–396 (1986).

2. S. J. Clough, A. F. Bent, Floral dip: a simplified method for Agrobacterium-mediated transformation of *Arabidopsis thaliana*. *Plant J.* **16**, 735–743 (1998).

3. J. Schindelin, *et al.*, Fiji: an open-source platform for biological-image analysis. *Nat Methods* **9**, 676–682 (2012).

4. U. K. Laemmli, Cleavage of structural proteins during the assembly of the head of bacteriophage T4. *Nature* **227**, 680–685 (1970).

5. F. Kuhnert, *et al.*, Rapid single-step affinity purification of HA-tagged plant mitochondria. *Plant Physiol.* **182**, 692–706 (2020).

6. T. Isaacson, *et al.*, Sample extraction techniques for enhanced proteomic analysis of plant tissues. *Nat Protoc* **1**, 769–774 (2006).

7. G. H. Krause, E. Weis, Chlorophyll fluorescence and photosynthesis - the basics. *Annu. Rev. Plant Physiol.* **42**, 313–349 (1991).

8. A. Bräutigam, D. Gagneul, A. P. M. Weber, High-throughput colorimetric method for the parallel assay of glyoxylic acid and ammonium in a single extract. *Anal. Biochem.* **362**, 151–153 (2007).

9. M. Schwaiger, *et al.*, Anion-exchange chromatography coupled to high-resolution mass spectrometry: A powerful tool for merging targeted and non-targeted metabolomics. *Anal. Chem.* **89**, 7667–7674 (2017).

10. C. Engler, *et al.*, A golden gate modular cloning toolbox for plants. *ACS Synth. Biol.* **3**, 839–843 (2014).
